# Adaptive Time-Dependent Priors and Bayesian Inference to Evaluate SARS-CoV-2 Public Health Measures Validated on 31 Countries

**DOI:** 10.3389/fpubh.2020.583401

**Published:** 2021-01-21

**Authors:** Hugues Turbé, Mina Bjelogrlic, Arnaud Robert, Christophe Gaudet-Blavignac, Jean-Philippe Goldman, Christian Lovis

**Affiliations:** ^1^Medical Information Sciences Division, Department of Radiology and Medical Informatics, University of Geneva, Geneva, Switzerland; ^2^Medical Information Sciences Division, Diagnostic Department, University Hospitals of Geneva, Geneva, Switzerland

**Keywords:** infectious diseases, reproductive number estimation, non-pharmaceutical interventions, Bayesian inference (BI), health sciences, epidemiology, SARS -CoV-2, public health

## Abstract

With the rapid spread of the SARS-CoV-2 virus since the end of 2019, public health confinement measures to contain the propagation of the pandemic have been implemented. Our method to estimate the reproduction number using Bayesian inference with time-dependent priors enhances previous approaches by considering a dynamic prior continuously updated as restrictive measures and comportments within the society evolve. In addition, to allow direct comparison between reproduction number and introduction of public health measures in a specific country, the infection dates are inferred from daily confirmed cases and confirmed death. The evolution of this reproduction number in combination with the stringency index is analyzed on 31 European countries. We show that most countries required tough state interventions with a stringency index equal to 79.6 out of 100 to reduce their reproduction number below one and control the progression of the pandemic. In addition, we show a direct correlation between the time taken to introduce restrictive measures and the time required to contain the spread of the pandemic with a median time of 8 days. This analysis is validated by comparing the excess deaths and the time taken to implement restrictive measures. Our analysis reinforces the importance of having a fast response with a coherent and comprehensive set of confinement measures to control the pandemic. Only restrictions or combinations of those have shown to effectively control the pandemic.

## Introduction

Since being first observed in Wuhan in late 2019, the outbreak of the 2019 SARS-CoV-2 virus is strongly affecting societies and economies. The transmission rate, pressure on the healthcare system and lack of effective treatment lead countries to take public health measures to limit the spread of the virus. The confinement measures range from banning gatherings to complete lockdowns and closing borders (1, 2). Additional measures include individual protection with various levels of mask wearing injunctions, and contact tracing with quarantine. This work has focused on developing reliable modeling approaches to evaluate the impact of public health measures. Our method is based on analyzing the reporting of European countries to evaluate the temporal influence of non-pharmaceutical interventions (NPIs) on the effective reproduction number *R*_*t*_. The aim of *R*_*t*_ is to quantify the number of secondary infections caused by an individual over the period during which this person is infectious. It is important to make the distinction between the effective and basic reproduction number. The basic reproduction number *R*_0_ refers to the evolution of the disease when the population is fully susceptible to the disease while *R*_*t*_ factors numerous parameters, such as the susceptible population, the transmission, the public awareness, the immunity acquired within the population, amongst others (3). *R*_*t*_ is a key parameter to evaluate the evolution of an epidemic. Any value below one indicates that the spread is decreasing, any value above one indicates that the spread is increasing in a given population. *R*_*t*_ allows a direct comparison of the epidemiologic profiles observed in different cohorts of population, such as specific risk factors driven cohorts or countries with distinct characteristics (such as population or testing methods). It allows thus to consider temporality and populational or cohorts characteristics. The spatial and populational (age, social activity) heterogeneity have been shown to play a role in the evolution of the pandemic as the *R*_*t*_ evolve differently across these different groups (4–6).

Numerous methods have been developed to compute *R*_*t*_ and its evolution over time (7) with the aim of identifying the most influential parameters and predicting the development of an epidemic in a given environment. Initial methods derived *R*_*t*_ from transmission model similar to the SIR model (8–12). In general, fitting deterministic model to incidence data has been shown to often results in large error which can however be solved by using stochastic model (13). The choice of the mechanistic transmission model requires assumptions about the epidemiology of the disease. For example, the presence/absence of a latency period will guide the choice between a SIR (Susceptible—Infected—Recovered) or SEIR (Susceptible—Exposed—Infected—Recovered) model. Recent studies tend to acknowledge the risk of asymptomatic transmission of COVID-19 although with a lower relative risk than transmission by symptomatic individuals (14) favoring the use of a SIR model. The latter model is parametrized through the transmission rate β and the rate of removal γ. One pitfall is that this model assumes a constant transmission rate, that is the infection probability distribution is constant over the period during which an individual is infectious. In addition, the SIR model requires to be fitted to the number of infections as well as the number of people either susceptible or who have recovered. However, the latter two variables, susceptible and recovered, are difficult to evaluate and will strongly be influenced by underreporting. Later models, including the Wallinga and Teunis approach (15), use a likelihood-based estimation procedure to reconstruct infection patterns. These methods have shown large variations when using daily data (16). Most approaches aiming at correcting these fluctuations appeared to be sensitive to smoothing parameters (16, 17). An additional method to mitigate these drawbacks that is very robust to underreporting was later developed (18). This method used Bayesian inference based on a transmission model which includes the infectivity profile to update the posterior distribution of *R*_*t*_ as more data become available.

Since the start of the COVID-19 pandemic, various studies have looked at the impact of public health interventions on the evolution of the pandemic at regional or national level. The first studies, on data from China, proving the impact of NPI strategies to reduce *R*_*t*_ used mechanistic transmission models to obtain *R*_*t*_ (19, 20), with the drawbacks described above associated with these models. Further studies focused on how state interventions prevented ICU capacity to be overwhelmed as well as their impact on fatalities in the UK (21), Germany (22, 23), and France (24). While these researches focused on individual country, a recent study aimed to demonstrate the impact of non-pharmaceutical interventions in 11 European countries (25). This study assumed that the impact of the measures was independent of their relative introduction. In addition, this study assumed *R*_*t*_ to be fixed between the different measures. However, a recent research shows that community changes also play a role in slowing the evolution of the virus (26).

When evaluating the impact of public health interventions, it is crucial to consider that there is a delay between the time of infection and the time at which a confirmed case or the death of an individual is reported. Even if we consider that NPIs have a direct impact on the rate of infections, there will be a delay between this change of infections and the time at which this change is observed through positives tests or the death of the individuals. The simplest method would consist in shifting the data backward in time by the mean of the distribution of interest that is the period from infections to the case being reported or the death of the individual. However, this method does not account for the uncertainty in the period of interest. A possible method to circumvent this issue consists in subtracting samples from the delay distribution to each observation. This method has been recently used to adjust reporting delays in the aim of evaluating the reproduction number of SARS-CoV-2 (25, 27) and was applied in our research. One drawback of the method is that as the mean and variance of the delay distribution increase, the resulting infections are smoothed over time potentially blurring discontinuities in the variation of *R*_*t*_ (28). Alternatively, the confirmed cases can be considered as the convolution of the infections with a delay period distribution. The process to obtain the time of infection can therefore be performed using a maximum-likelihood deconvolution method (29, 30). These methods build on techniques which were initially develop to correct AIDS data based on an iterative EM algorithm (31). A different approach aimed to jointly infer the infections and *R*_*t*_ (32). The drawback of this method is that it requires an hypothesis on the shape and change points of *R*_*t*_.

The aim of this work is to extend previous research estimating *R*_*t*_ and focusses on the effects of state interventions in 31 European countries. As the evolution of *R*_*t*_ is a function of at least three important parameters: the type of the restrictive measures; the effect of these measures and changes in behaviors with specific societal properties, and the size of various compartmental cohorts involved, we do not aim to quantify the effect of each measure. The restrictive measures and their effects are first considered to be independent across the different countries. We then compare their effects across the countries and aim to show how the combined interventions within a country and their temporality have influenced the spread of the virus, characterized by the evolution of confirmed cases, confirmed deaths, and excess deaths.

## Materials and Methods

The following section aims to describe the different steps of the analysis. The various data sources used in the analysis as well as required period distributions for SARS-CoV-2 are first introduced. Secondly, statistical methods to estimate *R*_*t*_ are formulated and lastly the method to evaluate the impact of NPIs is described.

### Data Sources and Availability

*R*_*t*_ is estimated using incidence data for confirmed cases and deaths published in the *COVID-19 Data Repository* (33).

The excess mortality was retrieved from Our World in Data, (34). The data are aggregated on a weekly basis along the average deaths observed for the same period between 2015 and 2019.

Data related to the period between a positive test and the death of an individual were retrieved from: Swiss Federal Office of Public Health (FOPH) (35). Data from FOPH on confirmed cases is used to evaluate the impact of different information sources.

Data regarding the various state interventions were retrieved from the *Coronavirus government response tracker* (OxCGRT) developed by the Blavatnik School of Government (36). The stringency index provided in this dataset tracks government's policies and interventions across different categories and provides a score between 0 and 100 evaluating the overall stringency of the measures taken in a given country (37). A stringency index of zero means no measure has been noted in this country, and a maximum score of 100, indicates a complete lock down. The stringency index is calculated as averages of the individual component indicators categorized in the following six groups: school closing, non-essential economic activities, public events, gatherings, stay at home policies, and restrictions on movements. For the “Stringency index” the sub-index score *I*_*j,t*_ is calculated for the 9 indicators as follows:

(1) $$\begin{array}{l} I_{j,t}=100\frac{v_{j,t}-0.5\left(F_{j}-f_{j,t}\right)}{N_{j}} \end{array}$$

With *N*_*j*_ being the maximum value of the indicator, *F*_*j*_ the indicator flag (whether the measure has or not a sectoral scope), *v*_*j,t*_ the recorded policy on the ordinal scale, and finally *f*_*j,t*_, being the recorded binary flag for that indicator. The full methodology, the variable values for computing the different scores are available on their github repository, along with the interpretation of each indicator (see Data Availability Statement for the exact reference). The evolution of the stringency index for the countries of interest can be found in Supplementary Figure 1.

A dataset which included the intersection of the data regarding the evolution of the confirmed cases and deaths as well as the data measuring the stringency index was available for 33 European countries. For our analysis, Russia and Ukraine were removed from our dataset as the reported daily deaths were still increasing for these two countries when we are interested in countries which have successfully contained the evolution of the pandemic before the 23rd of May 2020. We were therefore left with a set of 31 European countries. The full list of the countries included in the analysis is presented in the results sections. For the second part of the analysis which focused on the excess deaths observed in each country, the data were available for 19 countries.

### Determining Incubation Time, Onset to Confirmed, and Onset to Death Distributions

The proposed method allows to compute *R*_*t*_ without developing a transmission model and hence only requires a hypothesis on the infectivity profile or serial interval distribution. The infectivity profile is a probability distribution measuring the probability to infect an individual at a given time *s* after the infection of the primary case. This distribution is crucial to model the dynamic of the infections and the delay between the primary and secondary cases. The incidence on a given day can be estimated as follows:

(2) $$\begin{array}{l} E\left[I_{t}\right]=R_{t}\sum_{s=1}^{t}w_{s}I_{t-s} \end{array}$$

where *E*[•] is the expected value of a random variable, *I*_*t*_ is the incidence at time *t*, and *w*_*s*_ is the infectivity profile. The distribution of *w*_*s*_ for the SARS-CoV-2 virus was found to have a mean of 4.8 days and a standard deviation of 2.3 days (38).

Given the time at which the infection occurred is not available, the number of confirmed cases and deaths on a given day are used as proxies. A gamma distribution with a median incubation period at 4.4 days from confirmed infection and diagnosis outside of the epicenter of Hubei Province, China, based on official reports from governmental institutes was derived (39). The mean and deviation were then obtained by fitting a gamma distribution to the quantile derived in this study. The period between the onset of the symptoms and a case being confirmed in Switzerland, was estimated to 5.6 days (40).

The period between a case being reported as positive and the death of the individual was extracted from 1,430 cases provided by the Swiss Federal Office of Public Health (FOPH). Our result provides a distribution on a much larger dataset than the one built which used between 24 and 33 cases (39, 41). Three different distributions were tested: lognormal, Weibull and gamma with the Akaike Information Criterion (AIC) being used to identify the best distribution. This distribution was then combined with the incubation period (39) to obtain the period between onset and death shown in Table 1 along the other distribution periods where the onset refers to the symptom onset.

**Table 1:** Incubation, onset to confirmed and onset to death distributions where onset refers to symptoms onset.

**Period**	**Mean [days]**	**Standard deviation [days]**
Incubation (39)	4.6	1.9
Onset to confirmed (40)	5.6	4.2
Onset to death (our study)	15.3	8.0

From the latter period functions it is possible to calculate a posterior distribution of *R*_*t*_ based on the inferred infection dates extracted from the confirmed cases and deaths reported. For the daily cases declared (incidence), a shift following a gamma distribution between the defined cases (confirmed or dead) and the time of infection is randomly generated. For each case, the new date of infection is generated by subtracting the shift to the reported date. This procedure is performed iteratively with the mean of daily simulated number of infections stored. Using the latter period functions to estimate the infection occurrences allows to take into account the large variance in the cases reported by the health or political systems in the analyzed countries.

### Correcting the Number of Infections

In addition, the incidence for the most recent days are corrected (40) to factor delayed reporting:

(3) $$\begin{array}{l} {\bar{I}}_{t}=\frac{I_{t}}{{\hat{F}}_{l}} \end{array}$$

where ${\bar{I}}_{t}$ and *I*_*t*_ are, respectively, the corrected and initial incidence which took place on a given day. $\hat{F}$ is the cumulative distributive function of the period between an infection and a case being reported as positive or dead, *l* is the time between *t* and the last reported case so that ${\hat{F}}_{l}=\text{P}(X\leq l)$ where *X* is a random variable that is gamma distributed with mean and standard deviation described in Table 1 depending on the variable of interest and *P*(*X* ≤ *l*) is the probability that *X* is smaller or equal to *l*.

### Estimation of the Reproduction Number Using Bayesian Inference With Time-Dependent Priors

The method presented in this report is a variation of the one proposed by Cori et al. (18). Assuming the incidence at time *t*, *I*_*t*_, is Poisson distributed so that the likelihood of the incidence *I*_*t*_ given *R*_*t*_ and conditional on previous incidences *I*_0_, ⋯, *I*_*t*−1_:

(4) $$\begin{array}{l} P\left(I_{t}|I_{0},\cdots \;,I_{t-1},w,R_{t}\right)=\frac{{\left(R_{t}\Lambda_{t}\right)}^{I_{t}}e^{-R_{t}\Lambda_{t}}}{I_{t}!} \end{array}$$

with $\Lambda_{t}=\overset{t}{\underset{s=1}{\sum }}w_{s}I_{t-s}$ where *w*_*s*_ is the estimated infectivity profile.

The posterior of *R*_*t*_ conditional on previous incidences is:

(5) $$\begin{array}{l} P\left(R_{t}|I_{0},\cdots \;,I_{t-1,}I_{t},w\right)\propto P\left(I_{t}|I_{0},\cdots \;,I_{t-1},w,R_{t}\right)P\left(R_{t}\right) \end{array}$$

While the method developed by Cori et al. (18) assumes a constant gamma distribution for the prior distribution, the presented model takes advantage of the information gained in time by updating the prior distribution for each window with the previous posterior:

(6) $$\begin{array}{l} P\left(R_{t}\right)=P\left(R_{t-1}|I_{0},\cdots \;,I_{t-2},I_{t-1},w\right) \end{array}$$

The 95% CI is then derived by computing the 2.5% and 97.5% quantiles.

*R*_*t*_ based on the confirmed cases is reported up to 9 days before the last date at which results are available. This corresponds to the median time for confirmed cases to be reported. Using the same method, *R*_*t*_ based on the cases reported as dead is reported up to 19 days before the last day on which deaths were reported for a given country.

### Comparison of the Methods to Estimate *R*_*t*_ on Synthetic Data

In order to compare the proposed methods with the one developed by Cori et al. (18), a study on synthetic data was performed. Two scenarios which were initially used in the aforementioned research were used:

1. Constant reproduction number, *R*_*t*_ = 2.5
2. Sharp change in the reproduction number:
  $$\begin{array}{l} ◦R_{t}=\{\begin{array}{cc} 2.5, & t\leq 15days\\ 0.8, & t>15days \end{array} \end{array}$$

For each scenario, 100 simulations were performed. Ten cases were introduced at *t* = 0 *days*, with the incident cases *I*_*t*_ for the following 49 days being drawn from a Poisson distribution with mean equal to $R_{t}\overset{t}{\underset{s=1}{\sum }}I_{t-s}\;w_{s}$. An infectivity profile *w*_*s*_ with a mean of 4.8 and standard deviation of 2.3 days as introduced by Nishiura et al. (38) for the SARS-CoV-2 virus was used. *R*_*t*_ was then evaluated from the synthetic data using the method developed by Cori et al. (18) as well as the proposed method.

The impact of underreporting was simulated using a binomial distribution as performed in (18). For each day, the new incident cases $I_{t}^{*}$ were assumed to follow a binomial distribution:

(7) $$\begin{array}{l} I_{t}^{*}\sim Binomial\left(I_{t},\pi \right) \end{array}$$

where π is the reporting rate and was varied between 20 and 80% in steps of 20%. *R*_*t*_ was then evaluated on the simulated underreported data and compared to the simulated *R*_*t*_.

### Assessing NPIs' Impact on the Evolution of the Pandemic

The stringency index developed as part of the OxCGRT project (37) was used to assess the role of state interventions in controlling the pandemic. This index was compared with the evolution of *R*_*t*_, rather than the incidence of confirmed or dead cases. Using *R*_*t*_ helps comparing countries that have heterogeneous testing or reporting policies. While *R*_*t*_ is also subject to variations in these policies, it depends on the change within the country in confirmed and death cases, therefore allowing comparison between countries with different policies. For each country, the public health measures and the stringency index are analyzed when *R*_*t*_ estimates, based on the confirmed cases, dropped below one. The hypothesis is that it can help identifying the most efficient set of public health measures.

In order to assess the impact of taking restrictive measures early in the crisis, the time taken to introduce initial restrictive measures was compared to the period taken to control the epidemic. The time until the introduction of restrictive measure was defined as the period between the 5th death in a given country and the stringency index reaching a score of 35. The stringency index threshold at 35 corresponds to the lowest score observed when a country reached a *R*_*t*_ smaller than one which was observed for Andorra. The time required to control the epidemic was then defined as the period between the 5th death and *R*_*t*_, based on the confirmed cases, dropping below one.

Given that the confirmed cases and reported deaths are influenced by reporting policies, the analysis described above was supported by using the number of excess deaths. Following the same logic as for the previous analysis, the period between the 5th death and the stringency index reaching 35 was compared to the excess deaths experienced in each country. The excess deaths were calculated as:

(8) $$\begin{array}{l} Excess\;deaths=\\ \sum_{w}\frac{\;Deaths{|}_{Week\#w\;2020\;}-\;Average\;Deaths{|}_{Week\#w\;2015-2019\;}\;}{\;Average\;Deaths{|}_{Week\#w\;2015-2019\;}} \end{array}$$

where *w* represents for each country the weeks between the 5th death and the 23rd of May 2020. This alternative method to measure the impact of the different NPIs independently of the proposed method to compute *R*_*t*_ serves as a mean to support our conclusions.

## Results

### Evaluation of the Proposed Methods

**Figure 1:**
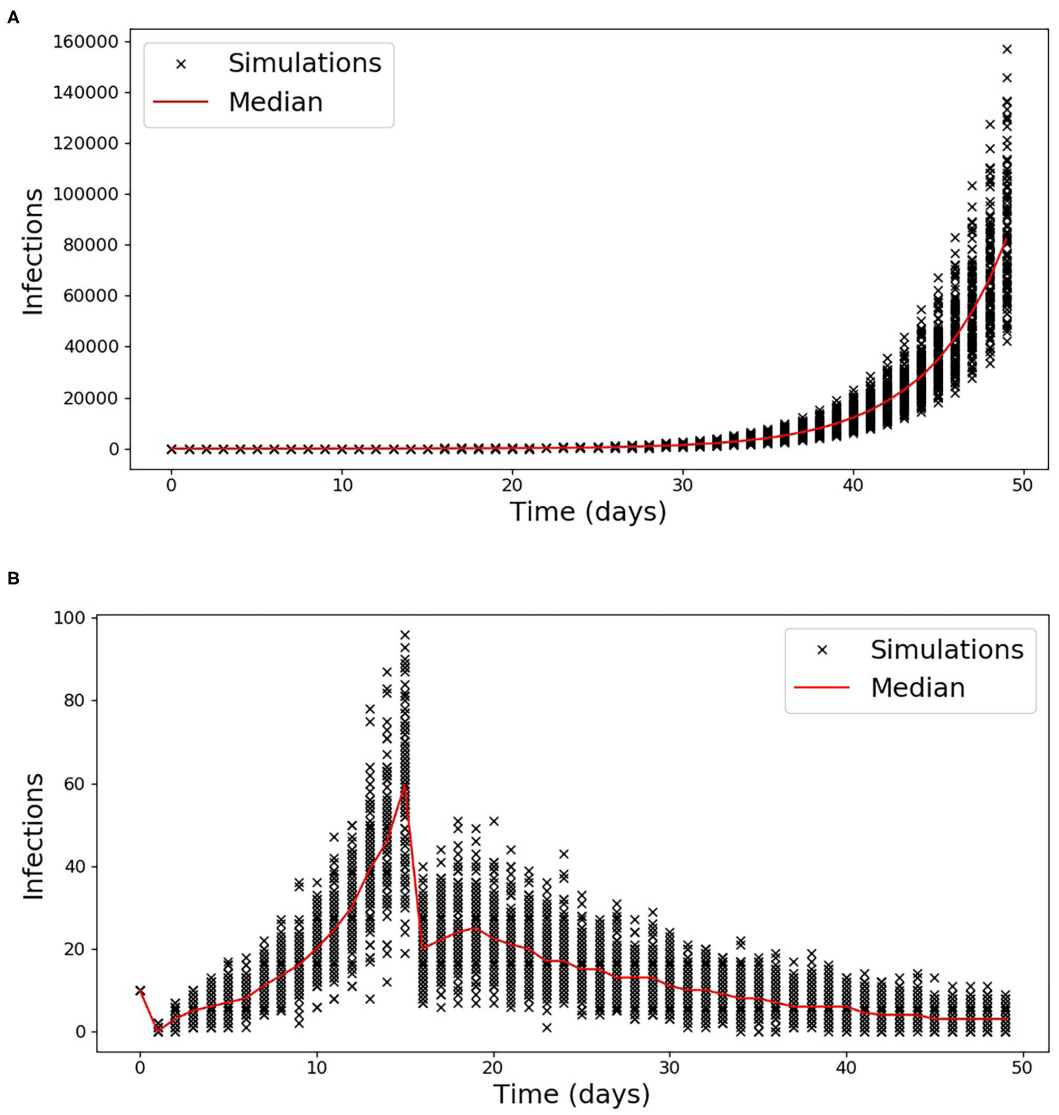
Simulated Incident cases for scenario 1 **(A)** and 2 **(B)**. The incident cases for each of the 100 simulations are reported with a cross while the median is indicated by the red line.

**Figure 2:**
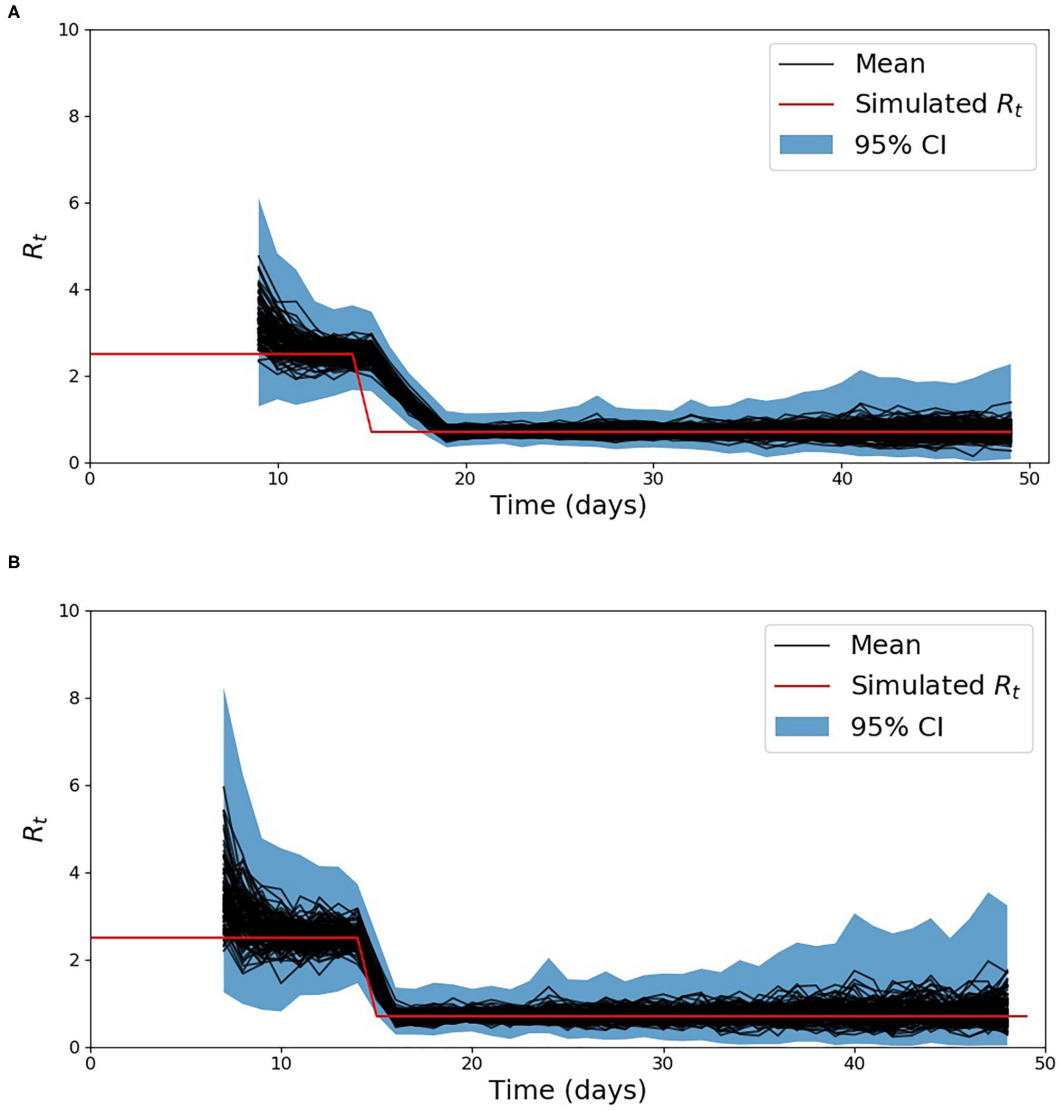
Estimation of *R*_*t*_ for scenario 2, using the baseline method developed by Cori et al. (18) **(A)** and the developed method **(B)**.

The simulated incident cases described in section Comparison of the methods to estimate *R*_*t*_ on synthetic data are presented in Figure 1 for the two scenarios used to validate the proposed methods. The *R*_*t*_ computed using the proposed method as well as the one from (18) for the first scenario are shown in Supplementary Figure 2, while the results for the second scenario which includes a discontinuity in the simulated *R*_*t*_ are shown in Figure 2. In order to compare the two methods, the average relative error was computed using the following equation:

(9) $$\begin{array}{l} Error=\frac{1}{l}\;\sum_{t=0}^{l}\frac{\left|R_{t}-\bar{R_{t}}\right|}{\bar{R_{t}}} \end{array}$$

where l is the number of days for which the computed *R*_*t*_ can be derived from the simulated incident cases and $\bar{R_{t}}$ is the simulated reproduction number over the same period *l*.

The computed average relative errors for the two scenarios and methods are presented in Table 2.

**Table 2:** Average relative error comparison between the proposed method and the one developed by Cori et al. (18) measured on synthetic data.

	**Average relative error [%]**		
	**Baseline method**	**Proposed method**	****Δ** [%]**
Scenario 1	1.81	0.87	−51.9
Scenario 2	17.7	9.01	−49.2

The *R*_*t*_ evaluated on the simulated underreported data following the method described in section Comparison of the methods to estimate *R*_*t*_ on synthetic data are presented for a reporting rate of 20, 40, 60, and 80% for the two scenarios in Supplementary Figures 3–8. The average relative errors for these simulations are shown in Table 3.

**Table 3:** Average relative error comparison between the proposed method and the one developed by Cori et al. (18) measured on the underreported synthetic data.

		**Average relative error [%]**		
**Underreporting rate **π****	**Scenario**	**Baseline method**	**Proposed method**	****Δ** [%]**
0.2	1	5.34	5.3	−0.75
	2	28.91	31.81	10.03
0.4	1	2.27	1.72	−24.23
	2	20.02	17.14	−14.39
0.6	1	2.06	1.23	−40.29
	2	19.3	12.1	−37.31
0.8	1	1.8	1.04	−42.22
	2	18.4	9.78	−46.85

The proposed method takes as input confirmed cases which can be provided by different sources (health or political systems). In Supplementary Figure 9, the reproduction number is estimated for Switzerland, with two different sources.

### Evaluating the Reproduction Number From Incidence Data of 31 Countries

The list of countries analyzed along dates characterizing the evolution of the epidemic and stringency index values are listed in Table 4 which is composed of four panels. This table summarizes our analysis performed by computing *R*_*t*_, based on the confirmed cases. The first panel includes the dates which were used to characterize the evolution of the pandemic in each country. The first column of this panel is the date at which the 5th death was observed, the 2nd one when the stringency index reached a value of 35 and the third one includes the date at which the country managed to control the epidemic by reducing *R*_*t*_, below one. The second panel shows the value of the stringency index when *R*_*t*_, was reduced below one. The third shows the period between the 5th death and the stringency index reaching 35 or *R*_*t*_ becoming smaller than one. The last panel includes the computed excess deaths. The same table with the data when *R*_*t*_ is evaluated on the reported deaths can be found in Supplementary Table 1.

**Table 4:** List of countries along dates characterizing the evolution of the epidemic (with R_t_ measured on the confirmed cases) and measured excess deaths in percent of the number of average death observed between 2015 and 2019.

	**Date**			**Value**	**Days from 5**^****th****^ **death to:**		
	**5th death**	**Str_**idx**_ > 35**	**R_t <1_**	**Str_**idx**_ when R_t <1_**	**Str_**idx**_ >35**	**R_t <1_**	**Excess death [%]**
Albania	26.03.2020	09.03.2020	31.03.2020	84	−17	5	
Andorra	29.03.2020	25.03.2020	24.03.2020	35	−4	−5	
Austria	19.03.2020	13.03.2020	22.03.2020	85	−6	3	7.5
Belgium	17.03.2020	14.03.2020	04.04.2020	81	−3	18	45
Bosnia and Herzegovina	29.03.2020	11.03.2020	01.04.2020	90	−18	3	
Bulgaria	28.03.2020	13.03.2020	29.03.2020	73	−15	1	
Croatia	29.03.2020	14.03.2020	26.03.2020	96	−15	−3	
Czechia	25.03.2020	11.03.2020	26.03.2020	82	−14	1	
Denmark	19.03.2020	11.03.2020	31.03.2020	72	−8	12	3.9
Estonia	02.04.2020	16.03.2020	27.03.2020	72	−17	−6	4.6
Finland	27.03.2020	16.03.2020	04.04.2020	60	−11	8	7.7
France	05.03.2020	13.03.2020	08.04.2020	91	8	34	23
Germany	13.03.2020	16.03.2020	26.03.2020	73	3	13	5.5
Greece	19.03.2020	12.03.2020	26.03.2020	84	−7	7	2.9
Hungary	22.03.2020	11.03.2020	08.04.2020	77	−11	17	0.2
Iceland	06.04.2020	16.03.2020	23.03.2020	54	−21	−14	
Ireland	23.03.2020	13.03.2020	09.04.2020	91	−10	17	
Italy	24.02.2020	22.02.2020	20.03.2020	92	−2	25	43
Luxembourg	21.03.2020	13.03.2020	22.03.2020	80	−8	1	17
Netherlands	13.03.2020	12.03.2020	05.04.2020	80	−1	23	34
Norway	18.03.2020	12.03.2020	23.03.2020	70	−6	5	2.6
Poland	22.03.2020	12.03.2020	05.04.2020	81	−10	14	2.8
Portugal	20.03.2020	16.03.2020	29.03.2020	82	−4	9	14
Romania	23.03.2020	09.03.2020	09.04.2020	87	−14	17	
Serbia	28.03.2020	15.03.2020	11.04.2020	100	−13	14	
Slovakia	15.04.2020	10.03.2020	13.04.2020	87	−36	−2	
Slovenia	26.03.2020	16.03.2020	24.03.2020	79	−10	−2	2.9
Spain	07.03.2020	10.03.2020	25.03.2020	72	3	18	55
Sweden	16.03.2020	29.03.2020	19.04.2020	46	13	34	29
Switzerland	13.03.2020	13.03.2020	21.03.2020	77	0	8	16
United Kingdom	10.03.2020	22.03.2020	08.04.2020	76	12	29	

*Str_idx_ denotes the Stringency index*.

**Figure 3:**
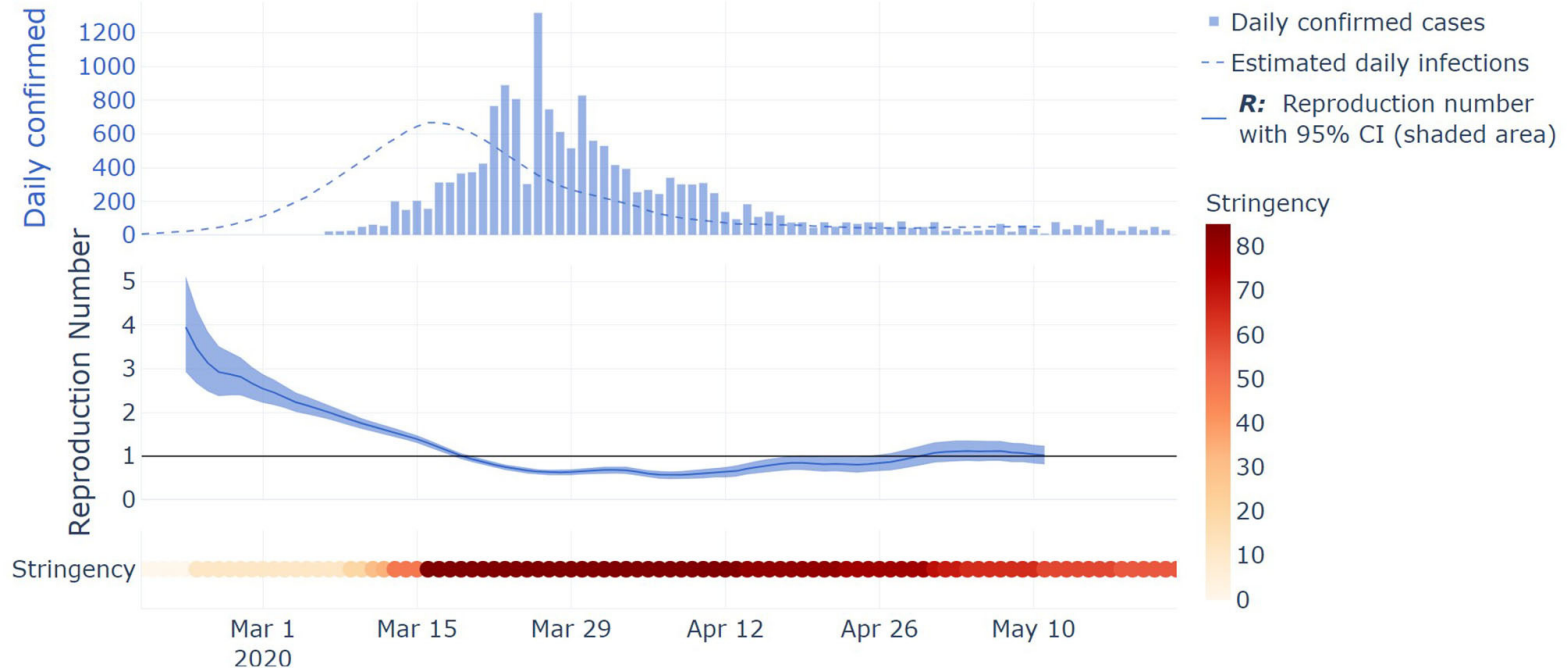
Illustration of key steps of the methodology on Austria case study. Top part: histogram of the daily confirmed cases, and inferred daily infections from daily confirmed cases and the derived period distributions displayed as a dashed line. Middle part: the mean estimated *R*_*t*_ is displayed as a full line, along with its 95% CI displayed as a shaded area, with *R*_*t*_ being estimated from the inferred infections. Bottom part: the evolution of the stringency index is displayed with a colorbar changing toward dark red as the stringency score goes toward its maximum value of 100, through the period of interest (from the date of the 5^th^ death up to the 23rd of May).

As a case study, the evolution of *R*_*t*_ in Austria is shown in Figure 3. Figure 3 aims to illustrate the different steps of the analysis and will be used for the discussion. In the top part, the daily confirmed cases are shown as a histogram. From these daily confirmed cases and the derived period distributions, the inferred daily infection are displayed as a dashed line. In the middle part, the mean estimated *R*_*t*_ is displayed as a full line, along with its 95% CI as a shaded area, with *R*_*t*_ being estimated from the inferred infections. In the bottom part, the evolution of the stringency index is displayed with a colorbar changing toward dark red as the stringency score goes toward its maximum value of 100, through the period of interest (from the date of the 5th death up to the 23rd of May). Different interesting phases of the pandemic are shown in the Austrian example depicted in Figure 3. Firstly, *R*_*t*_ started to decline before the introduction of restrictive measures between March 13th and 17th, and this reduction was intensified by a combination of NPIs which sums into a high stringency index score. *R*_*t*_ then plateaued at around 0.65 during the lockdown and has been oscillating around one up to the end date of our analysis (23rd of May). This last phase shows the emergence of localized clusters.

**Figure 4:**
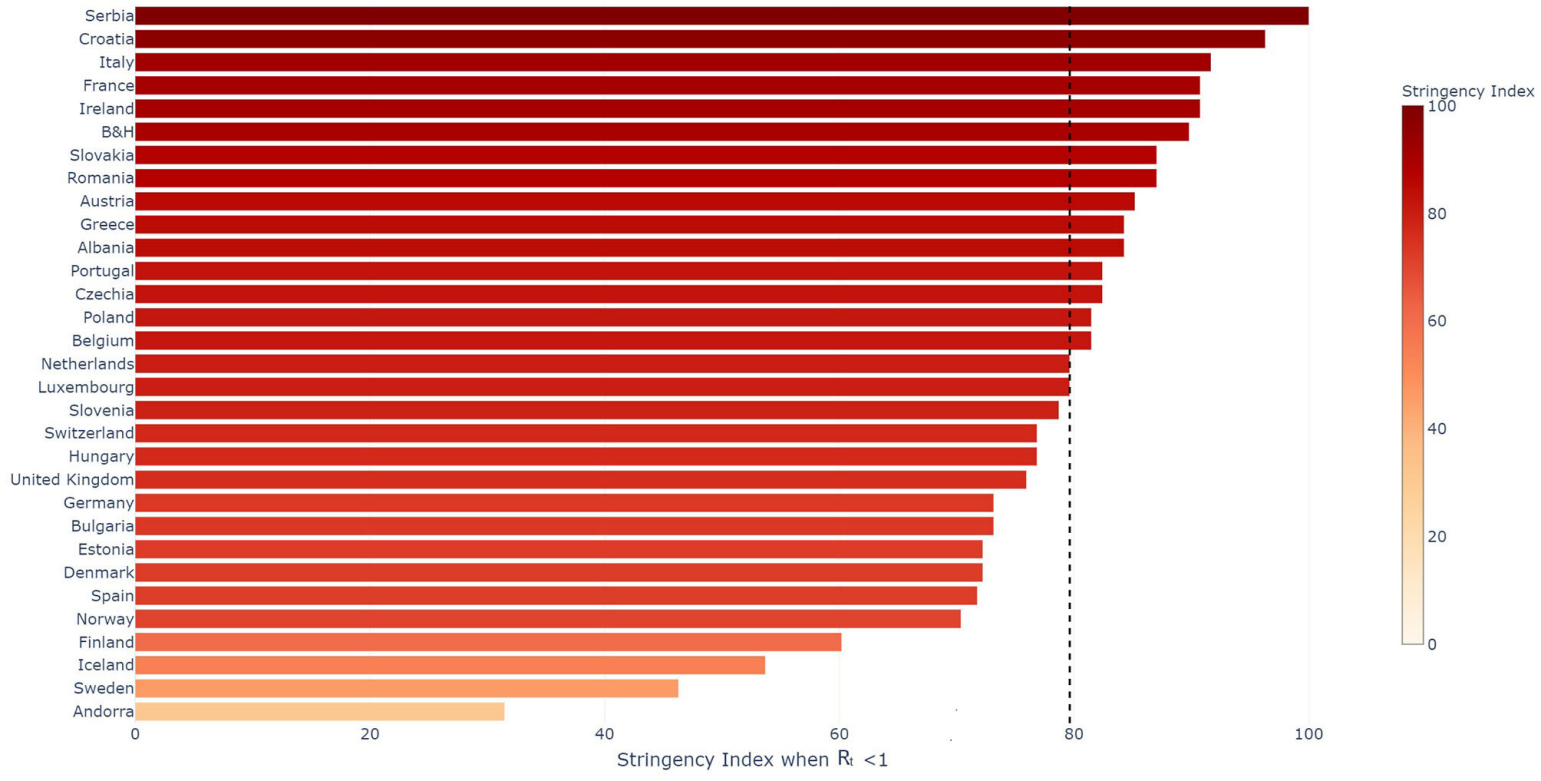
Stringency Index per country when *R*_*t*_ evaluated on the confirmed cases reduced below 1. The median value for the set of countries presented in the figure is indicated with the vertical black dotted line.

**Figure 5:**
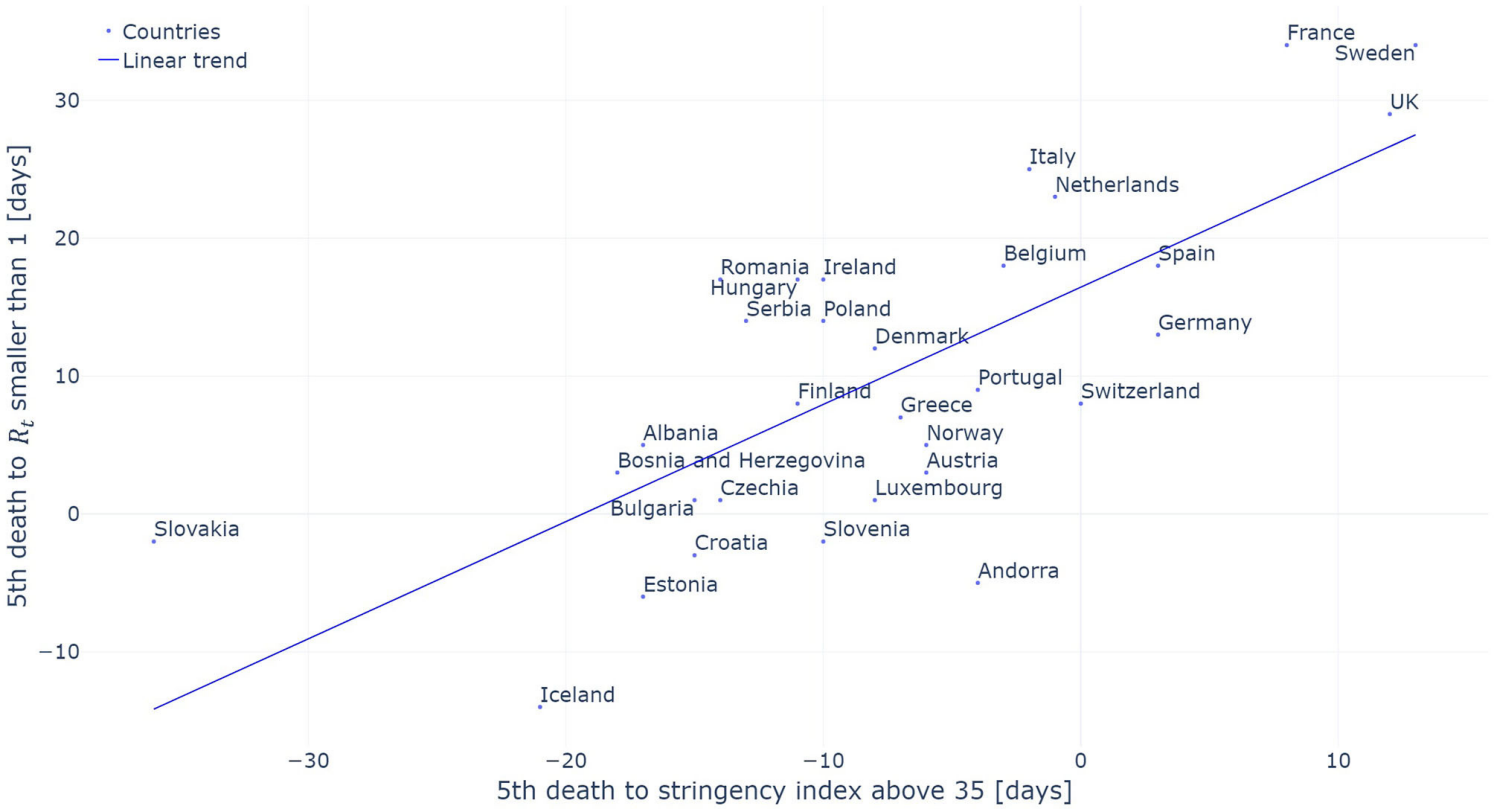
Period required to contain the epidemic (*R*_*t*_ smaller than one) evaluated on the confirmed cases as a function of the period between the 5th death and the introduction of initial restrictive measures (stringency index above 35). The linear trend is added for reference.

When countries managed to reduce their *R*_*t*_ estimated on the confirmed cases below one, they had a mean stringency index of 79.6 out of 100 with a standard deviation of 14.3. The individual stringency indices for each country are presented in Figure 4. When *R*_*t*_ dropped below one, the median severity of the measures along their individual severity out of 100 for each category defined in the OxCGRT dataset was the following: (a) School closed (100/100); (b) Non-essential economic activities closed (100/100); (c) Public events were canceled (100/100); (d) Gathering of more than 10 people banned (100/100); (e) Mandatory at home policy with minimal exceptions (67/100); (f) Movements in the country were restricted (100/100). Figure 5 shows the time from the 5th death to *R*_*t*_ reducing below one against the time from the 5th death to the date where the stringency reached 35. A Pearson correlation coefficient of 0.722 was found between the two variables.

This analysis was repeated for the *R*_*t*_ estimated on the reported deaths. A gamma distribution with a mean and a standard deviation equal, respectively, to 9.7 and 6.73 days was found, using the AIC criterion, to best fit the data from a case being confirmed to its death. The distribution along the extracted data are shown in Supplementary Figure 10. The AIC for the different distribution are summarized in Supplementary Table 2. This distribution was used to estimate the *R*_*t*_ on the confirmed deaths. A Pearson correlation coefficient of 0.338 was obtained between the two variables, that is the time between the 5th death and the stringency index reaching 35 and the time between the 5th death and the *R*_*t*_ reducing below one. The results for this analysis are presented in Supplementary Figures 11, 12.

**Figure 6:**
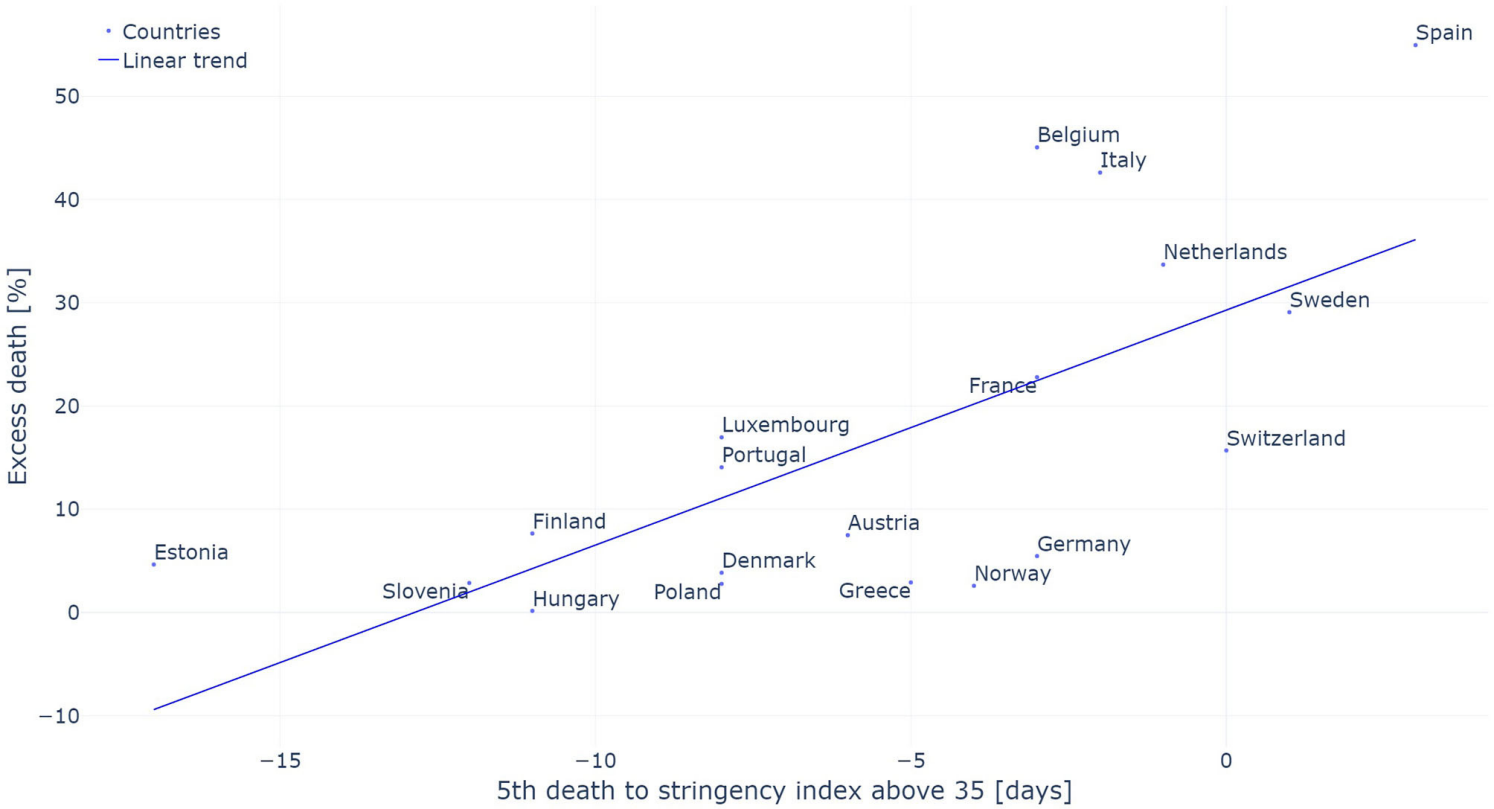
Excess death measured in percent of the number of average death observed between 2015 and 2019 as a function of the period between the 5th death and the introduction of initial restrictive measures (stringency index above 35). The linear trend is added for reference.

The comparison between the level of excess deaths observed in a given country and the time between the 5^th^ death and the stringency index reaching 35 are presented in Figure 6. A Pearson correlation of 0.684 was observed between these two variables.

## Discussion

### Evaluation of the Proposed Method to Estimate *R*_*t*_

The method developed to estimate the effective reproduction number *R*_*t*_ is based on the method developed by Cori et al. (18). This method only requires the infectivity profile and an initial assumptions of the basic reproduction number *R*_0_ used to initialize the prior. The difference and main advantage of the proposed method is that we are less reliant on the initial assumptions of *R*_0_. While (18) assumes the prior is fixed in time, we constantly adapt it with new data. As seen in Supplementary Figure 2, for a constant reproduction number both methods, the baseline and the proposed method converge toward the simulated value of 2.5. The similarity between the two methods on this scenario is also reflected in the average relative error presented in Table 2. Both methods have a low error, but the proposed method reduces the average error by around 1%. This reduction is mainly due to its faster rate of convergence toward the start of the simulated data. The difference between the two methods are more visible in the 2nd scenario which simulate a discontinuity in *R*_*t*_. This discontinuity aimed to simulate the extreme case where the introduction of a given NPI would have a direct effect on *R*_*t*_. As seen in Figure 2, the developed method tracks the sharp change in *R*_*t*_ arising on day 15 much more closely than the baseline method. As a result the average relative error over the simulations reduces from 17.7% with the baseline method to 9.0% with the developed method. This result is expected given that the distribution's prior is updated with the most recent data, while in the method proposed by Cori et al. (18), only the posterior evolves.

The baseline method was shown by its authors to be robust to underreporting (18). Given it is a known issue in the current pandemic and it was even more so toward the start of the pandemic, it was important to verify than the proposed methods retained this beneficial characteristic. As described in section Comparison of the methods to estimate *R*_*t*_ on synthetic data, underreporting was simulated on the synthetic data. As shown in Table 3, the developed method overperformed the baseline one in all simulated cases, except the 2nd scenario with a reporting factor of 20%. This error mainly arises from the incident cases which lies at the end of the simulated periods with only one or two incident cases being simulated over the last 15 days. Over all simulations which replicates underreporting (Supplementary Figures 3–8), the proposed method has a larger confidence interval when *R*_*t*_ is estimated on very small incident cases.

### Challenges in Estimating *R*_*t*_ on Real Data

As it is very difficult at the beginning of an epidemic to correctly evaluate *R*_0_ (42), it is important to update the prior as more data become available. In the future, our method will therefore be generalizable to new epidemic and provide reliable data at the start of the epidemic by being less reliant on the initial estimation of *R*_0_. However, as previous methods developed to estimate *R*_*t*_, our method is sensible to change in testing policy within a given country. It is also important to note that as there is a delay between the infection of an individual and the individual testing positive or dying, the *R*_*t*_ measured today reflects the evolution of the pandemic shifted in the past by the distribution of the period between the infection and the case being confirmed or the death of the individual. Models aiming to correct this delay have been initially developed to correct the data following the delay between a positive test and the test being reported (43) in order to allow real-time tracking of epidemics. More recently, Nowcasting methods using hierarchical Bayesian model have been used to provide reliable and up-to-date estimate of the *R*_*t*_ (44).

Confirmed cases and deaths are widely available in the public domain, but to estimate the infection dates, the incubation period and the period between the onset of the symptoms and the person having a positive test or the death of the individual is required. The incubation period was initially derived on Chinese cases (39) and it was assumed that this property is intrinsic to the virus and is therefore relevant for European countries. The period between the symptoms onset and a case being confirmed has been derived on Swiss patient (40). The period between the symptoms onset and the death of the patient was derived on Chinese data (39), but this period was not available for European patients. Based on 1,430 Swiss cases, we found this period to have a mean of 15.3 days compared to 16.3 days in Linton et al. (39). It was then assumed that this period was relevant for the European countries included in our study. All the periods distribution used for the rest of the analysis are summarized in Table 1.

Impact of data sources have been qualitatively evaluated for Switzerland. *R*_*t*_ has been separately estimated on data from the international repository of JHU (33) and the national repository of FOPH (35) for the same period of time (Supplementary Figure 9). The average relative error equation (9) between the two estimated *R*_*t*_ is 6%. This value is relatively low compared to the changes in the reported cases. As an example, *R*_*t*_ dropped below one for the first time for both estimates on the 18th of March, even though on the exact same day, the confirmed new cases were reported to be, respectively, 328 and 1,211, for JHU and FOPH sources. As visible in Supplementary Figure 9 in the appendix, the method seems to mitigate reporting inaccuracies, by providing an *R*_*t*_ with very similar trend.

### Impact of NPIs

Our analysis shows that when *R*_*t*_, based on the confirmed cases, reduced below one, the median severity of the measures for each category was important with a median stringency index of 79.6 out of 100. In addition, the standard deviation of the index, which is equal to 14.3, shows that most countries required measures with similar intensity achieved through different combinations of NPIs. It is not possible to determine the impact of each individual measure as most countries took them in different order and often a given country took multiples ones at the same time, but the high stringency index reinforces the central idea that only important combinations of NPIs allow to control the pandemic. This finding is consistent with the findings presented in (23) where it is shown that initial NPIs managed to reduce the *R*_*t*_, but that only a full contact ban reduced it below one. It is interesting to analyze the measure individually, not to determine their individual impact, but to determine which set of measures country had put in place when they successfully controlled the epidemic. If we look at the median restrictions when countries managed to control the epidemic, they were all at their maximum level apart from some exceptions on the closing of public transport as well as people being allowed to go out of with minimal daily exceptions. The two categories which had the strongest restrictions were the restrictions on public events and the school closing. All countries required canceling public events apart from Sweden and Andorra which only recommended to cancel them. One limitation of the dataset used in this analysis is that it does not measure whether people have to wear mask either in public transport or in all closed environments. It would be important to include those data as more countries are introducing this type of measures to prevent the resurgence of the virus. Also some NPIs could have a higher impact on the mortality, without having a significant impact on *R*_*t*_ evaluated on the confirmed cases. Lastly, the adherence of the population to NPIs is not taken into account here, and is definitely an important parameter to assess their impact on the spread of the pandemic within a country.

Our analysis also looked at the timing of NPIs introduction with the results presented in Figure 5. A strong correlation (Pearson coefficient of 0.722) between the time at which NPIs were introduced and the time at which a country managed to reduce *R*_*t*_ below one was found. This correlation indicates that countries which introduced NPIs early on manage to control the evolution of the pandemic within a shorter time frame. The use of the 5th death as a starting date allows to take into account that the pandemic did not start at the same time in all the countries analyzed in this study. The United Kingdom can serve as an interesting example. The UK had initially planned to build “targeted herd immunity” delaying the introduction of restrictive measure. As a result of this delay, the UK was only able to contain the epidemic 29 days after the 5th death occurred in the country when the median time for the countries included in our analysis was of 8 days. There are three outliers in our analysis being Andorra, Sweden and Iceland. Sweden has decided not to introduce a complete lockdown and stands with one of the highest daily death incidence in Europe [May 23rd: Sweden−5.34 deaths per million people per day; other European countries analyzed 0.82 on the same day (34)]. In the preceding analysis, no delay between the application of a measure and its effects on the reproduction number was taken into account. By doing so, the aim is to measure the timing between the introduction of the given measures and its effect on the *R*_*t*_ irrespectively of the behavioral impact it has on the inhabitants who might anticipate the introduction of the measures or inversely take some time to adapt to the introduced measures.

The analysis was replicated using *R*_*t*_ computed on the deaths linked to a SARS-COV2 infections and the data can be found in Supplementary Figures 11, 12. Similarly to the results presented above, countries had a median stringency index of 81.48 when they managed to reduce the *R*_*t*_ computed on deaths below one. It is interesting however to note that the analysis between the introduction of the NPIs and the time at which the *R*_*t*_ reduced below one showed much poorer correlation, Pearsons correlation factor of 0.338, compared to the same analysis on the confirmed cases. A critical limitation when analyzing the evolution of *R*_*t*_ evaluated on reported deaths is that the large variance in the distribution between the onset of the symptoms and the deaths of an individual spreads the retrieved infections. As a results, it becomes very difficult to detect sharp changes in *R*_*t*_ induced by the introduction of NPIs. This effect is similar to the effect of increasing the variance of the incubation period which was shown to decrease the ability to detect changes in *R*_*t*_ (18). The chosen method retrieves the infections dates by subtracting a shift drawn from the distribution of interest. The latter can effectively be seen as a convolution of the confirmed and death cases with the inverse distribution of the corresponding shift, hence spreading the retrieved in time compared to the true level of infections. Using a deconvolution method to retrieve the date of infections instead of the chosen method could improve the detection of changepoints in the trend.

The excess death observed in each country was compared to the timing of the introduction of the NPIs. This analysis has two main benefits. First, it allows to measure the impact of NPIs independently of the estimated *R*_*t*_ and its associated drawbacks described previously. Second, it allows to compare the size of the pandemic in each country without any bias introduced by changes in reporting policy withing a given country which impacts the *R*_*t*_. Such bias would include a rapid increase in the number of tests being performed as tests become more widely available. A Pearson correlation factor of 0.684 between these two variables indicates that countries which took restrictive measures earlier observed lower excess deaths. This high correlation between the timing at which NPIs were introduced and the level of excess death confirms the idea that the *R*_*t*_ evaluated on the confirmed deaths is not appropriate to evaluate the impact of these measures. One bias introduced by using the level of excess deaths to assess the impact of NPIs is that excess deaths will be larger in countries with older populations for a given penetration of the virus in the population as the elderly are much more vulnerable to the virus (45, 46).

A drawback of considering the evolution in the different countries at a national level and not at a regional one is that the heterogeneity of the spread of the virus is disregarded. To evaluate not only the effects of NPIs but also the resurgence of localized clusters, whose identification will be critical to avoid new waves, it is important to look where the cases are located at a more local level. There is therefore a trade-off where *R*_*t*_ is more reliable when evaluated on a larger amount of cases, but less representative as it does not take into account local disparities. Given the greater risk for older population to die or be hospitalized, it would also be interesting to assess the impact of different NPIs across different age groups.

## Conclusion

The proposed method to estimate the effective reproduction number *R*_*t*_ has been shown to be less reliant on the initial assumptions of *R*_0_ and to effectively improve the modelization of discontinuities in *R*_*t*_ which could be for example observed near the introduction of NPIs. The developed method was subsequently used to analyze the impact of NPIs on 31 European countries. It was first demonstrated that during the first semester of 2020, most European countries had to implement important restrictions to control the pandemic. Our analysis was further extended to show that early introduction of NPIs shortened the time required to control the evolution of the pandemic. The latter correlation was validated by highlighting a direct correlation between early adoption of restrictive measures and a reduction in the excess deaths.

Our study on the impact of health measures focused on European countries but can be extended to other countries for which data on the daily incidence as well as the NPIs taken on a given day are available. To extend this study to a larger set of countries, it would however be necessary to adapt the period between the onset of the symptoms and a case being confirmed or the death of a patient. However, while a sensitivity analysis would be required to assert the influence of variations in the different period distributions, the relatively small difference between the periods derived in Switzerland and in China (6.3%) in regards to the incertitude on the other parameters (daily incidence, infectivity profile) lets us believe that this factor is likely to play a marginal role if our analysis was to be extended to more countries.

Additional data could help refining our conclusions. First, we could add hospitalizations data as those would not be influenced by change in testing policies within a given country. In addition, looking at *R*_*t*_ within the different age groups could improve our understanding of the impacts of the different NPIs on these various groups. This information would be crucial to develop effective health policies protecting the most vulnerable while provoking minimal disruptions to the society and the economy.

## Data Availability Statement

Publicly available datasets were analyzed in this study. The excess death was retrieved from: Max Roser, Hannah Ritchie, Esteban Ortiz-Ospina, and Joe Hasell, Coronavirus Pandemic (COVID-19), Our World in Data, https://ourworldindata.org/coronavirus [accessed on 24/05/2020]. Data related to the period between a positive test and the death of an individual were retrieved from: Swiss Federal Office of Public Health (FOPH), Cas confirmés en laboratoire: distribution géographique, https://covid-19-schweiz.bagapps.ch/fr-1.html [accessed on 06/05/2020] R_*t*_ was estimated using incidence data for confirmed cases and death published in the COVID-19 Data Repository by Johns Hopkins University (JHU CSSE), https://github.com/CSSEGISandData/COVID-19 [accessed on 23/05/2020]. Data regarding the various state interventions were retrieved from the Coronavirus government response tracker (OxCGRT) developed by the Blavatnik School of Government, Oxford University, https://github.com/OxCGRT/covid-policy-tracker [accessed on 23/05/2020].

## Ethics Statement

Ethical review and approval was not required for the study on human participants in accordance with the local legislation and institutional requirements. Written informed consent from the participants' legal guardian/next of kin was not required to participate in this study in accordance with the national legislation and the institutional requirements.

## Author Contributions

HT performed the analysis of the data as well as the redaction of the article. MB contributed to the analysis as well as the redaction of the article. AR reviewed the method used to analyze the data. CG-B extracted the data used for the analysis. J-PG reviewed the article. CL initiated the project and the research question and contributed to the redaction of the article. All authors contributed to the article and approved the submitted version.

## Conflict of Interest

The authors declare that the research was conducted in the absence of any commercial or financial relationships that could be construed as a potential conflict of interest.
