# Supplementary material for: Adaptive Time-Dependent Priors and Bayesian Inference to Evaluate SARS-CoV-2 Public Health Measures Validated on 31 Countries
Source: Front Public Health. 2021 Jan 21;8:583401. doi: 10.3389/fpubh.2020.583401 (PMC7862946; doi:10.3389/fpubh.2020.583401)
Supplement: Supplementary file 1 [file Table_1.DOCX]

Supplementary Material

# Supplementary Data

Two csv documents, data_confirmed.csv, data_death.csv, can be found as part of the supplementary material. Each csv file includes the following columns:

- Country
- date-index
- Rt_min: Lower bound of the 95% CI interval for the computed $R_{t}$
- Rt_mean: Mean computed $R_{t}$
- Rt_max: Upper bound of the 95% CI interval for the computer $R_{t}$
- Retrieved_incidence: The retrieved infections for a given day

The analysis is done both on the confirmed cases and deaths with the results included in the corresponding file.

# Supplementary Figures


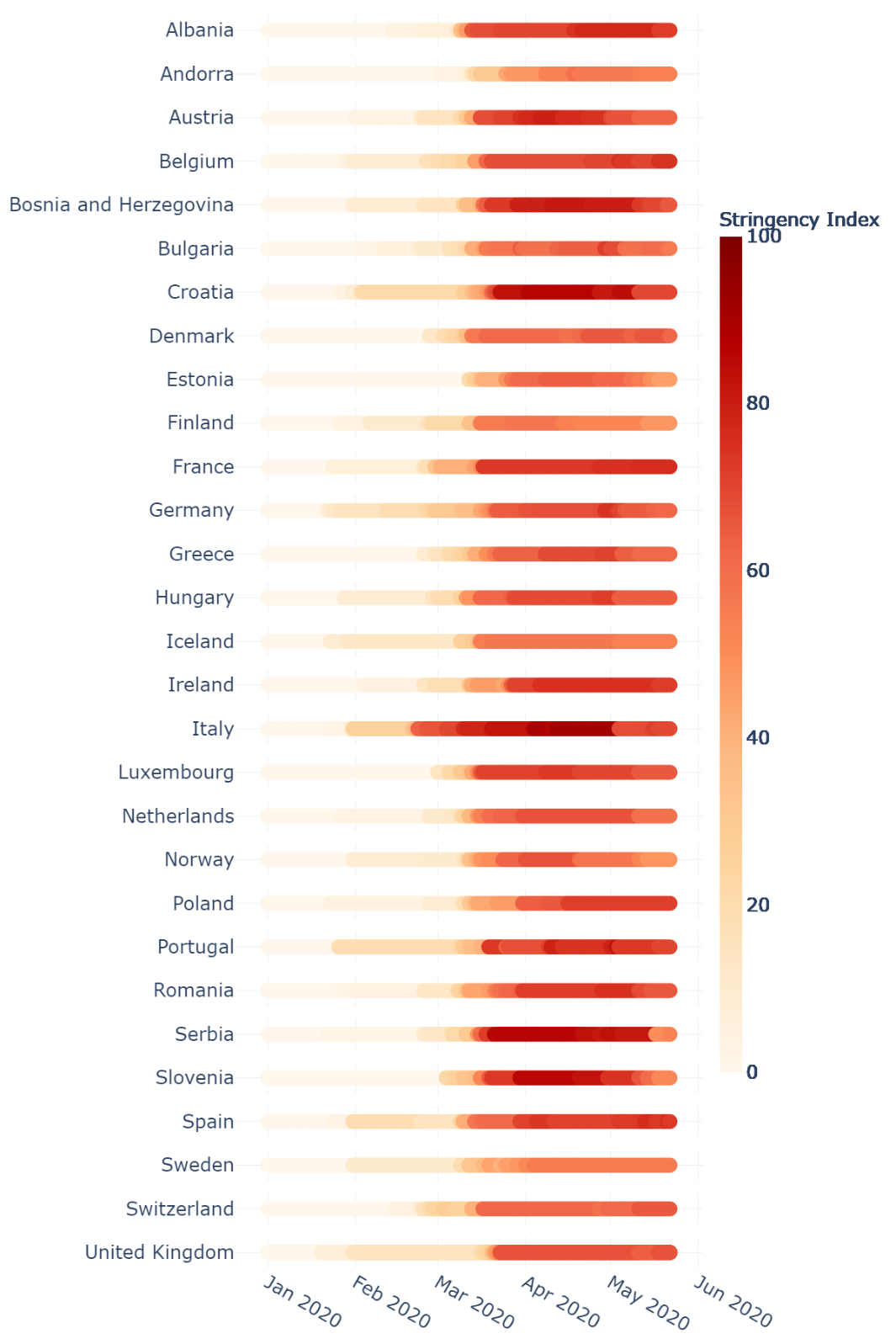


**Supplementary Figure 1:** The evolution of the stringency index for the countries of interest is displayed with a colorbar changing towards dark red as the stringency score goes towards its maximum value of 100, through the period of interest (from the date of the 5th death up to the 23rd of May)

| 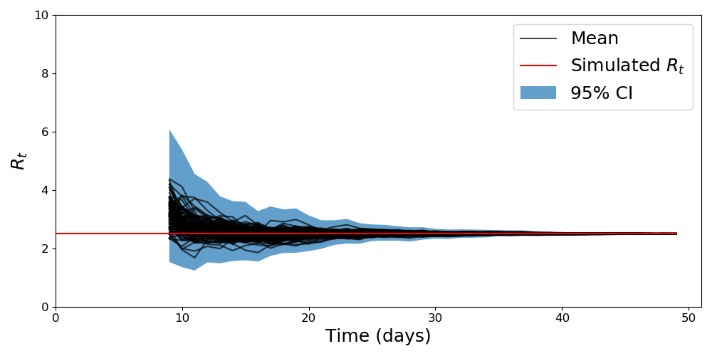 | 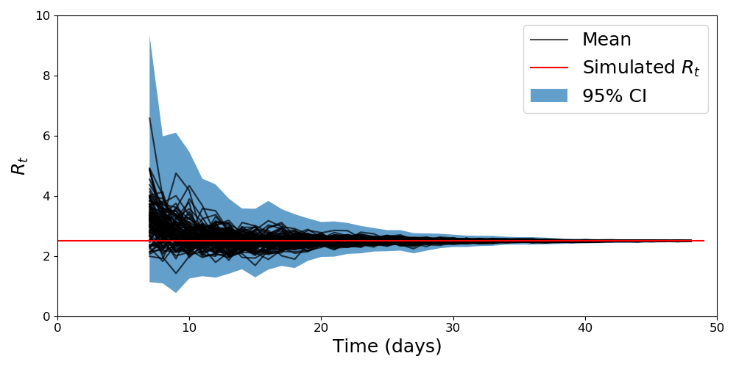 |
| --- | --- |
| (a) | (b) |

**Supplementary Figure 2:** Estimation of $R_{t}$ for scenario 1, using the baseline method developed by (Cori et al. 2013) (a) and the developed method (b)

| 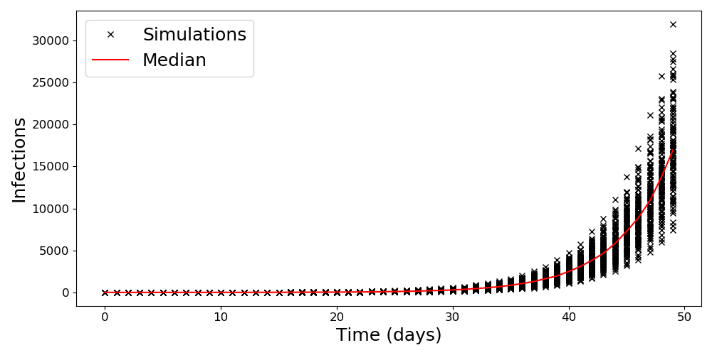 | 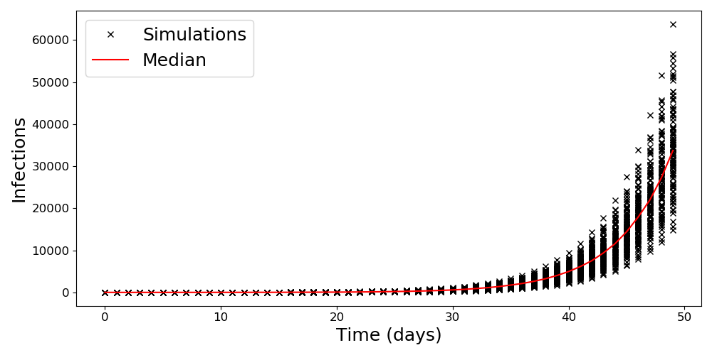 |
| --- | --- |
| (a) | (b) |
| **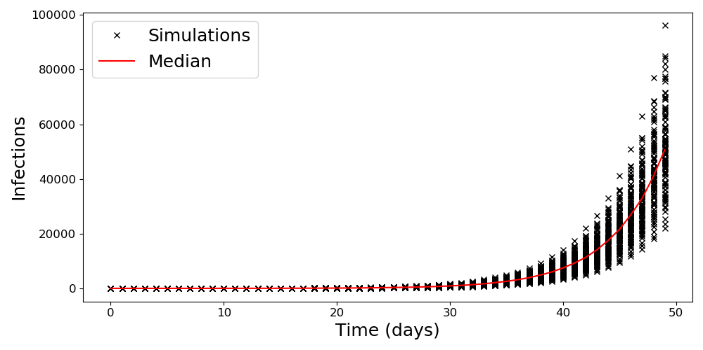** | **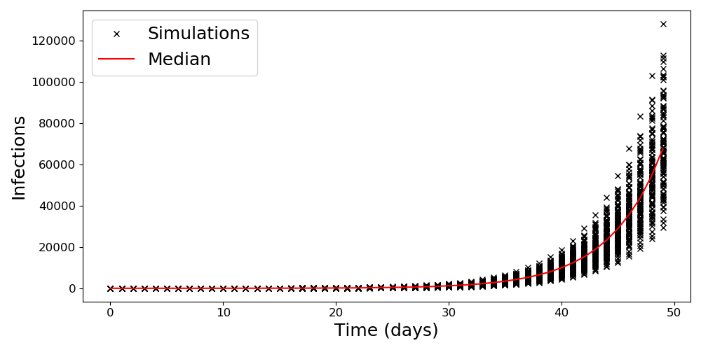** |
| (c) | (d) |

**Supplementary Figure 3:** Simulated Incident cases for scenario 1 with underreporting. The incident cases for each of the 100 simulations are reported with a cross while the median is indicated by the red line. The incident cases are simulated with a reporting rate of a) 20 %, b) 40%, c) 60%, d) 80%

| 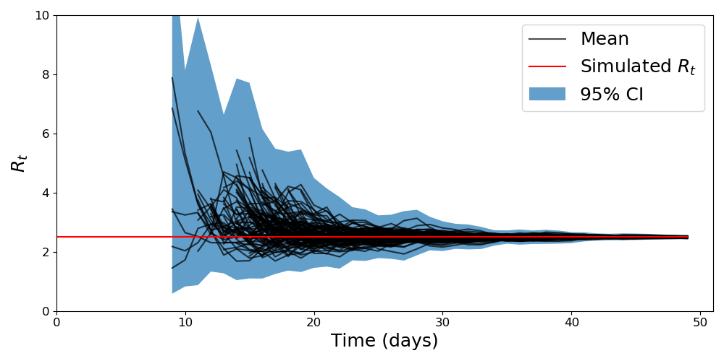 | 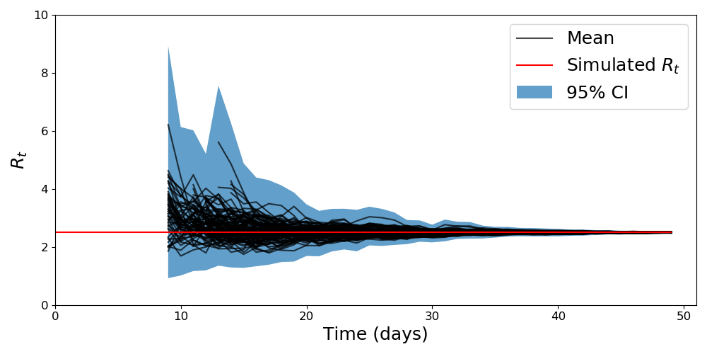 |
| --- | --- |
| (a) | (b) |
| **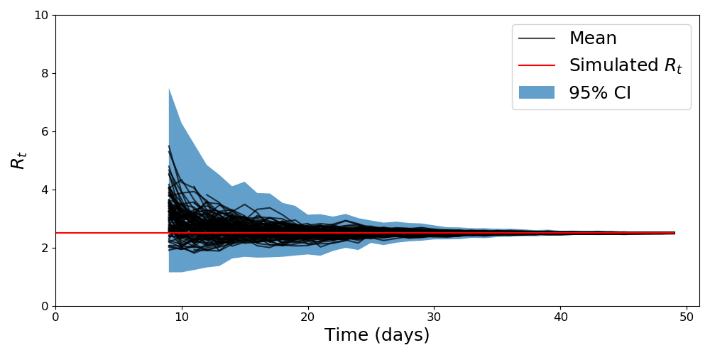** | **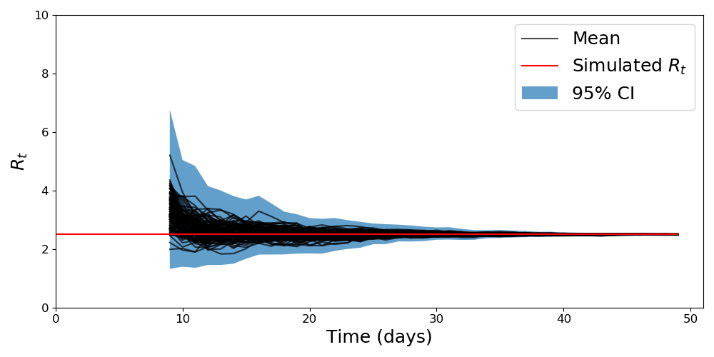** |
| (c) | (d) |

**Supplementary Figure 4:** Estimated $R_{t}$ for scenario 1 using the baseline method developed by (Cori et al. 2013) with a reporting rate of a) 20 %, b) 40%, c) 60%, d) 80%

| 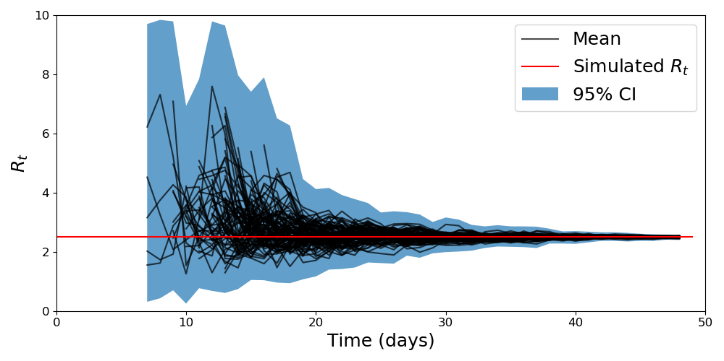 | 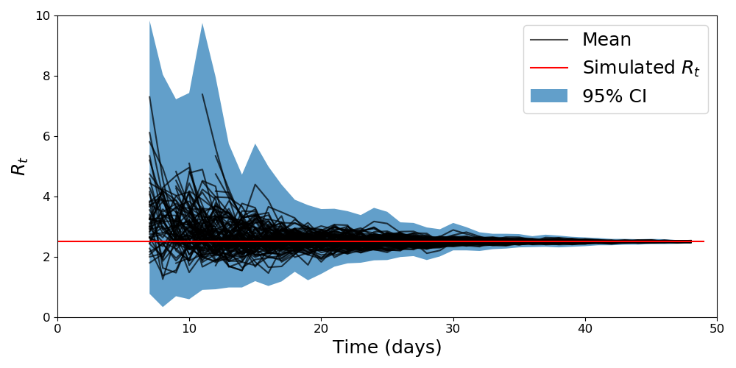 |
| --- | --- |
| (a) | (b) |
| **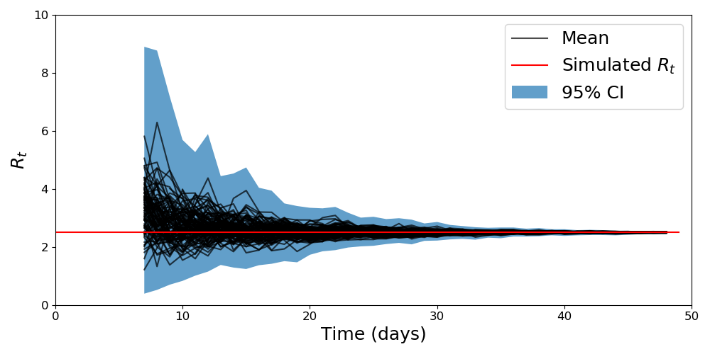** | **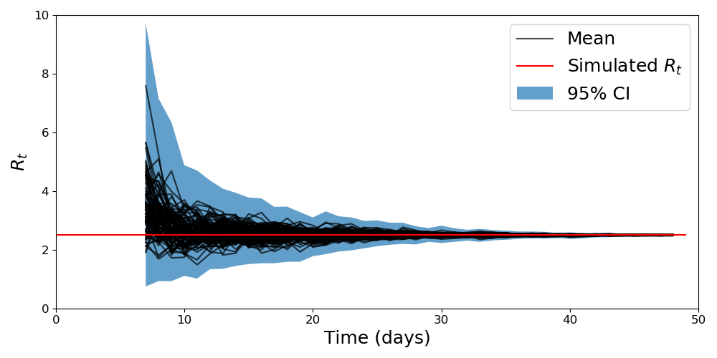** |
| (c) | (d) |

**Supplementary Figure 5:** Estimated $R_{t}$ for scenario 1 using the proposed method with a reporting rate of a) 20 %, b) 40%, c) 60%, d) 80%

| 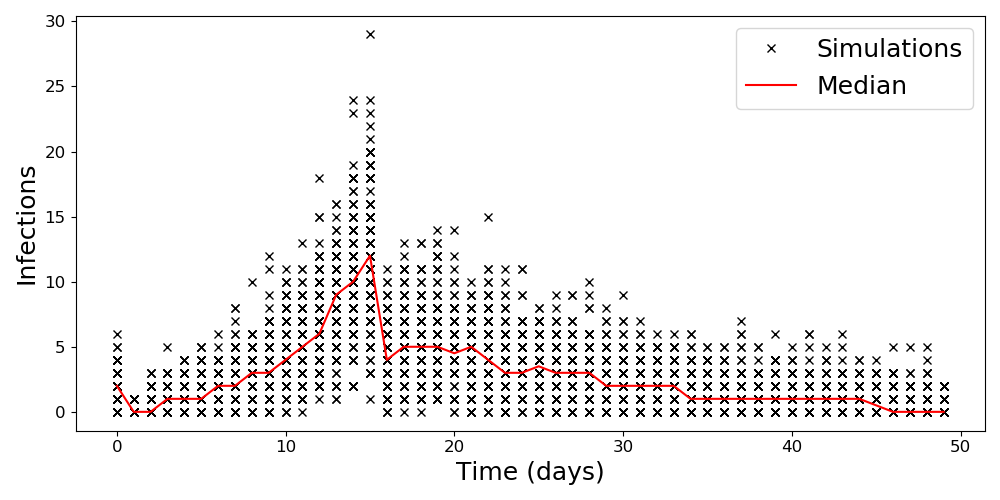 | 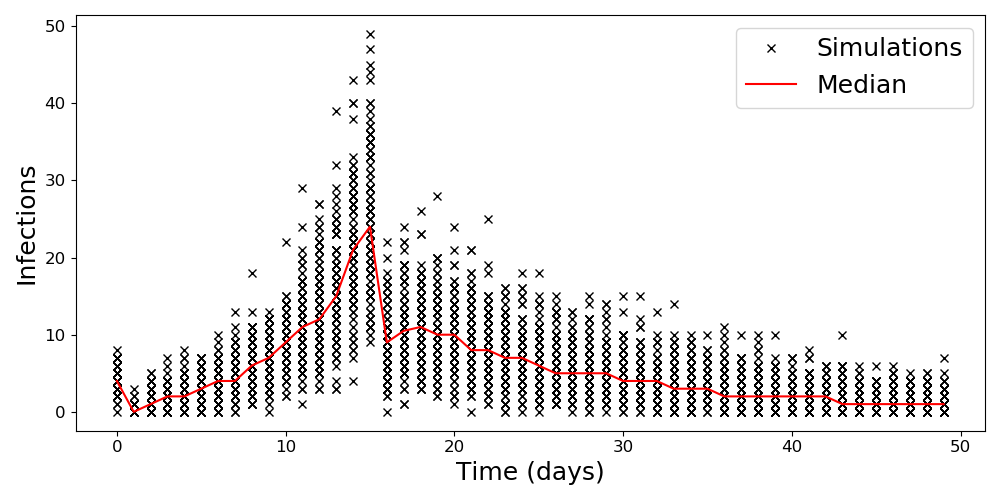 |
| --- | --- |
| (a) | (b) |
| **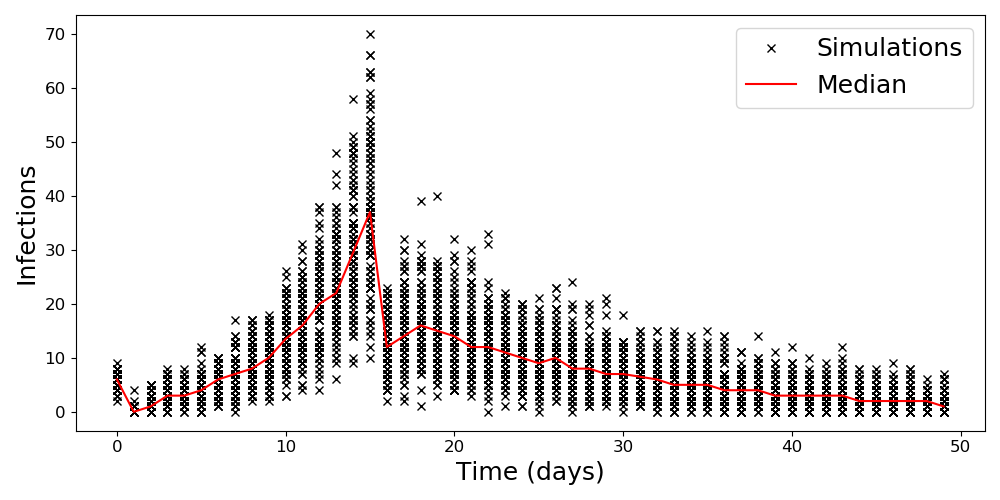** | **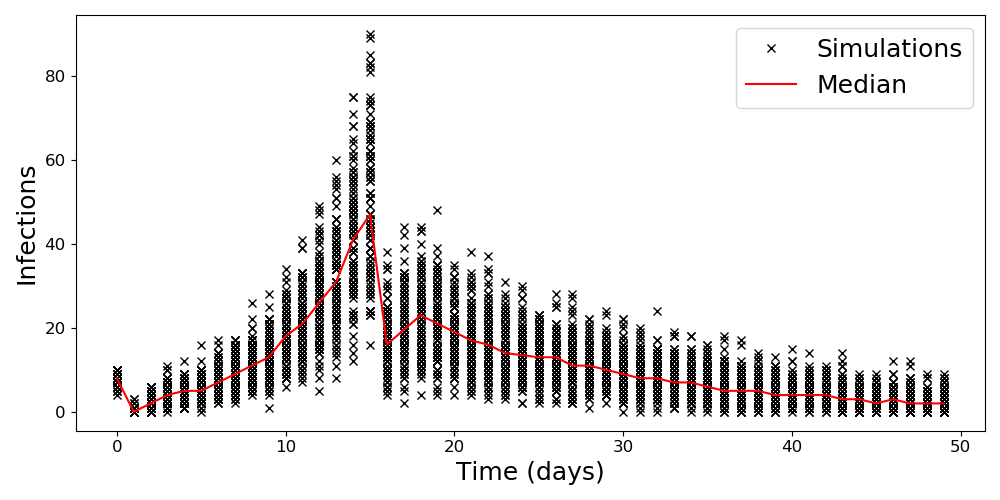** |
| (c) | (d) |

**Supplementary Figure 6:** Simulated incident cases for scenario 1 with underreporting. The incident cases for each of the 100 simulations are reported with a cross while the median is indicated by the red line. The incident cases are simulated with a reporting rate of a) 20 %, b) 40%, c) 60%, d) 80%

| 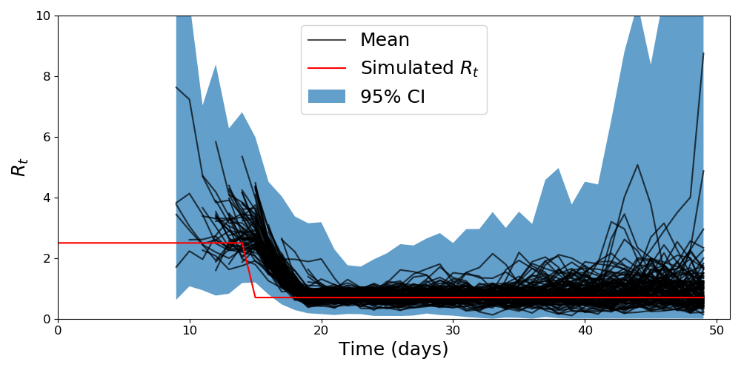 | 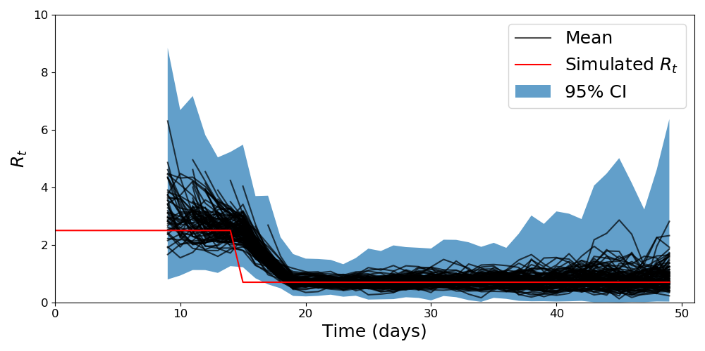 |
| --- | --- |
| (a) | (b) |
| **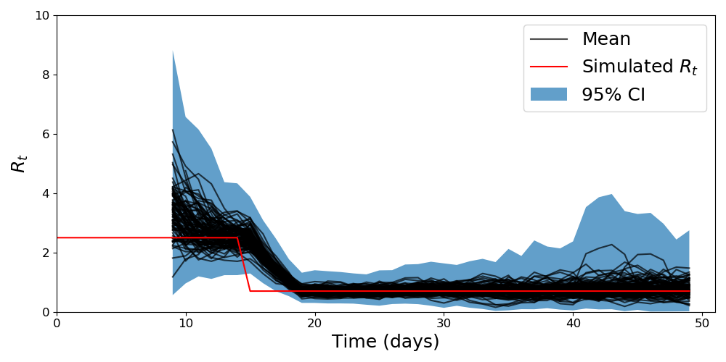** | **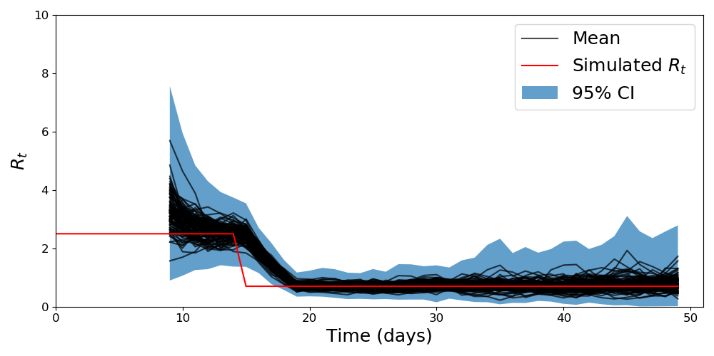** |
| (c) | (d) |

**Supplementary Figure 7:** Estimated $R_{t}$ for scenario 2 using the baseline method developed by (Cori et al. 2013) with a reporting rate of a) 20 %, b) 40%, c) 60%, d) 80%

| 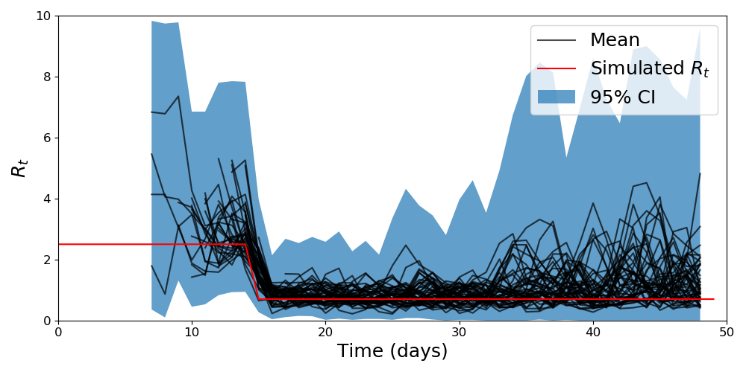 | 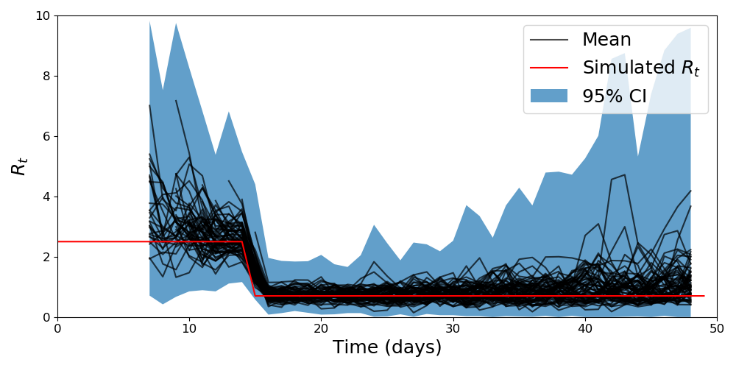 |
| --- | --- |
| (a) | (b) |
| **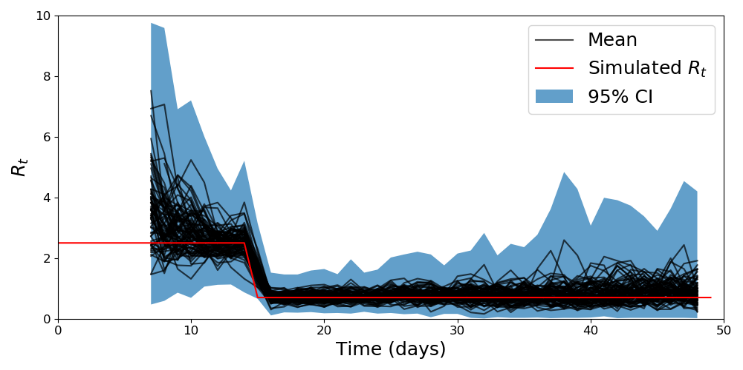** | **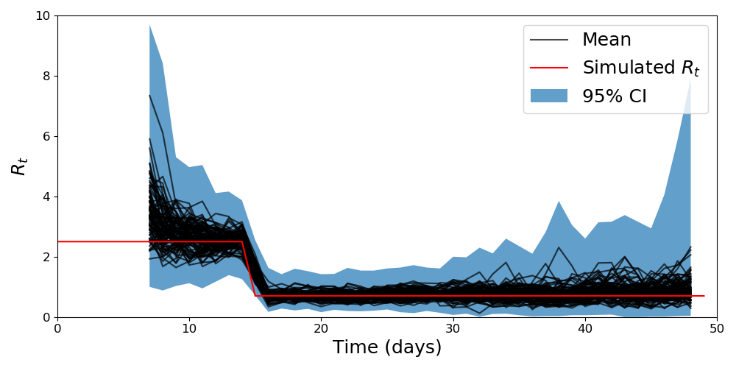** |
| (c) | (d) |

**Supplementary Figure 8:** Estimated $R_{t}$ for scenario 2 using the proposed method with a reporting rate of a) 20 %, b) 40%, c) 60%, d) 80%


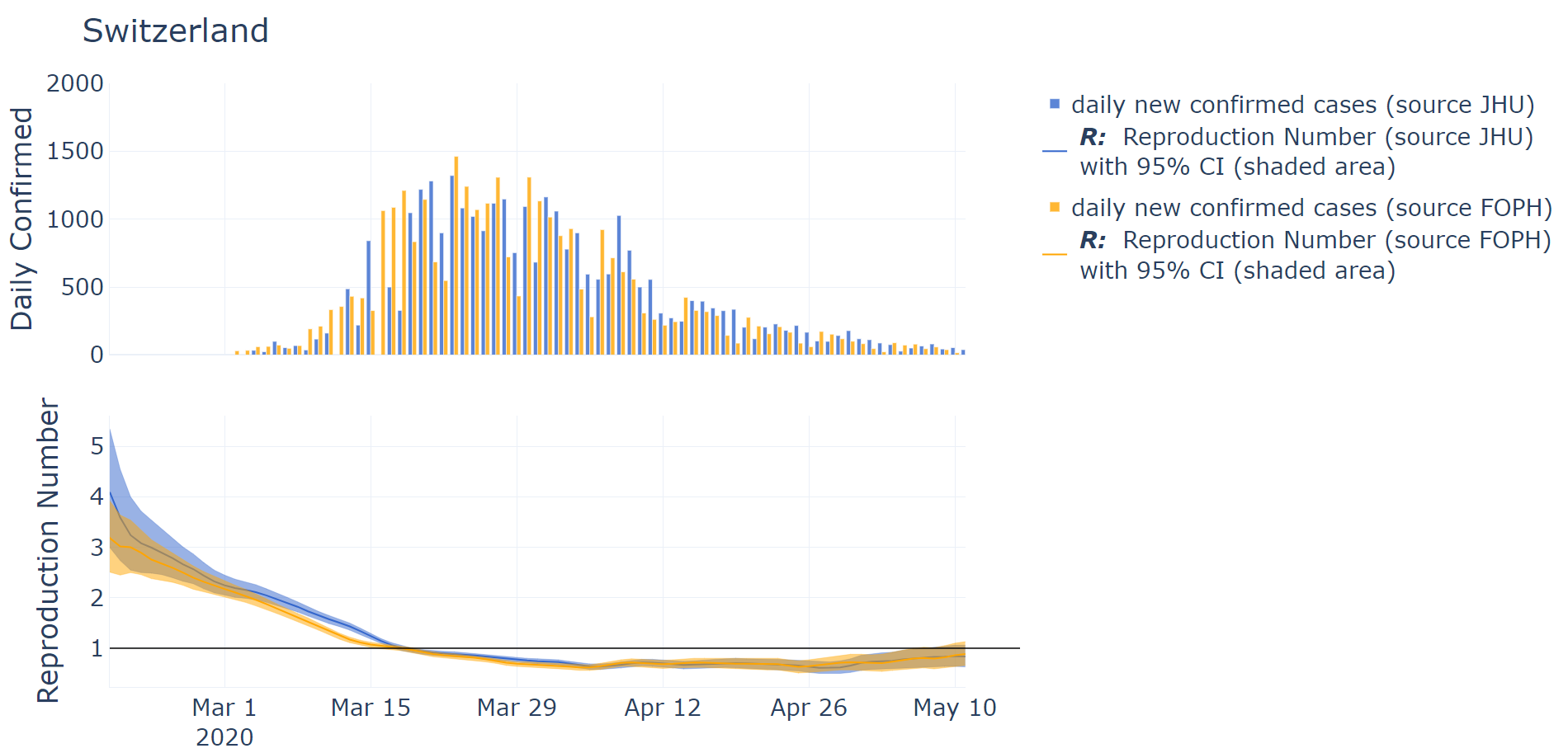


**Supplementary Figure 9:** Comparison of the confirmed cases and estimation of $R_{t}$ using data from JHU (depicted in blue) and FOPH (depicted in orange) (see data availability statement for reference).Top part: histogram of the daily confirmed cases. Bottom part: the mean estimated $R_{t}$ is displayed as a full line, along with its 95% CI displayed as a shaded area, with $R_{t}$ being estimated from the daily confirmed cases.


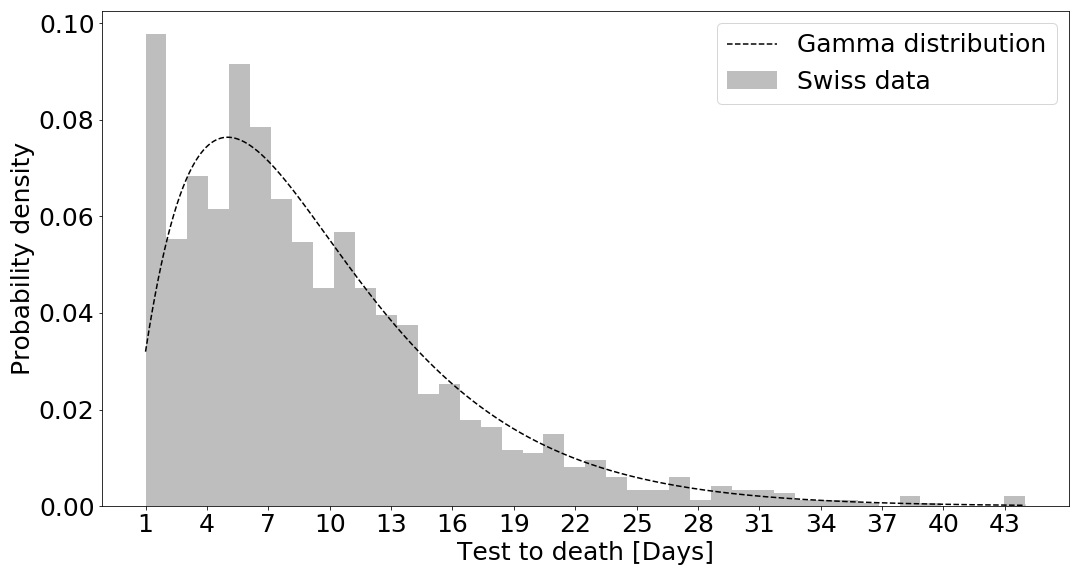


**Supplementary Figure 10:** Gamma distribution of the period in days between a positive test being reported and the time of death of the patient.


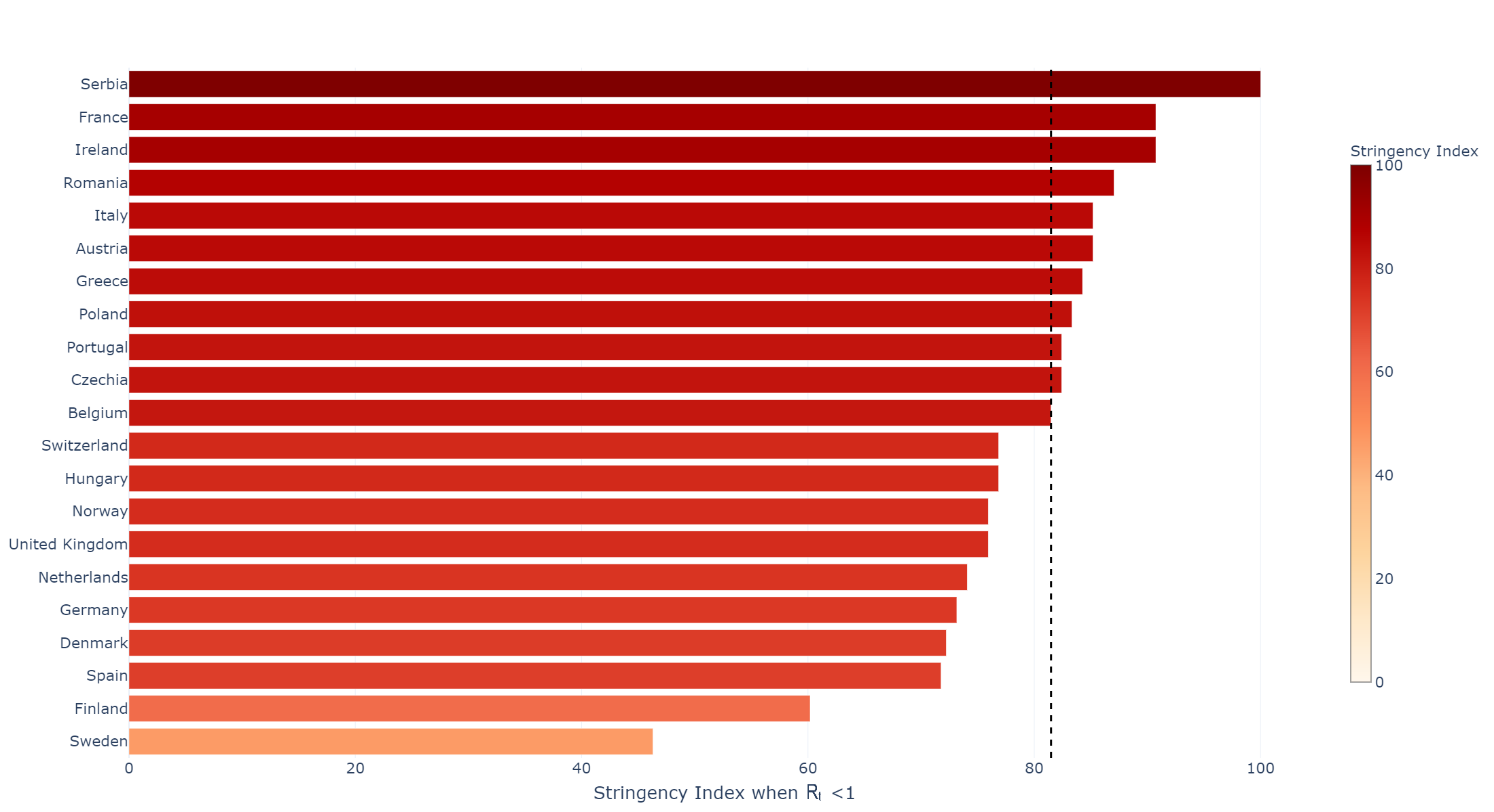
**Supplementary Figure 11:** Stringency Index per country when $R_{t}$ evaluated on the confirmed deaths reduced below 1. The median value for the set of countries presented in the figure is indicated with the vertical black dotted line.


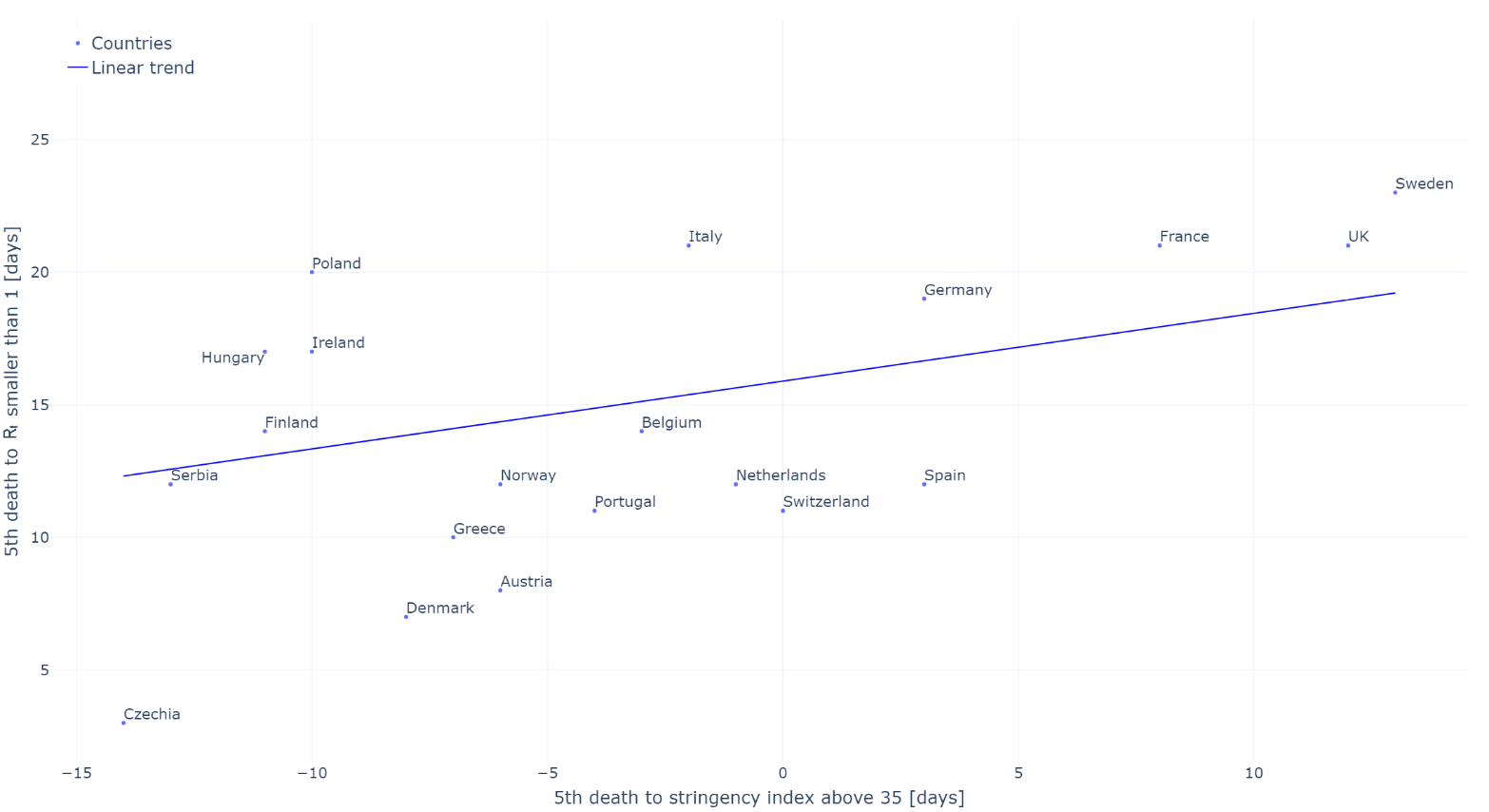


**Supplementary Figure 12:** Period required to contain the epidemic ($R_{t}$ smaller than one) evaluated on the confirmed death as a function of the period between the 5^th^ death and the introduction of initial restrictive measures (stringency index above 35). The linear trend is added for reference.

# Supplementary Tables

**Supplementary Table 1:** List of countries along dates characterizing the evolution of the pandemic. Str_idx_ denotes the Stringency index and the $R_{t}$ is measured on the confirmed deaths.

|  | Date | | | Value | Days from 5^th^ death to: | |
| --- | --- | --- | --- | --- | --- | --- |
|  | 5th death | Str_idx_ > 35 | $R_{t}$ <1 | Str_idx_  when | Str_idx_ >35 | $R_{t}$ <1 |
|  |  |  |  | $R_{t}$ <1 |  |  |
| Albania | 26.03.2020 | 09.03.2020 | 31.03.2020 | 84 | -17 |  |
| Andorra | 29.03.2020 | 25.03.2020 | 24.03.2020 | 35 | -4 |  |
| Austria | 19.03.2020 | 13.03.2020 | 22.03.2020 | 85 | -6 | 8 |
| Belgium | 17.03.2020 | 14.03.2020 | 04.04.2020 | 81 | -3 | 14 |
| Bosnia and Herzegovina | 29.03.2020 | 11.03.2020 | 01.04.2020 | 90 | -18 |  |
| Bulgaria | 28.03.2020 | 13.03.2020 | 29.03.2020 | 73 | -15 |  |
| Croatia | 29.03.2020 | 14.03.2020 | 26.03.2020 | 96 | -15 |  |
| Czechia | 25.03.2020 | 11.03.2020 | 26.03.2020 | 82 | -14 | 3 |
| Denmark | 19.03.2020 | 11.03.2020 | 31.03.2020 | 72 | -8 | 7 |
| Estonia | 02.04.2020 | 16.03.2020 | 27.03.2020 | 72 | -17 |  |
| Finland | 27.03.2020 | 16.03.2020 | 04.04.2020 | 60 | -11 | 14 |
| France | 05.03.2020 | 13.03.2020 | 08.04.2020 | 91 | 8 | 21 |
| Germany | 13.03.2020 | 16.03.2020 | 26.03.2020 | 73 | 3 | 19 |
| Greece | 19.03.2020 | 12.03.2020 | 26.03.2020 | 84 | -7 | 10 |
| Hungary | 22.03.2020 | 11.03.2020 | 08.04.2020 | 77 | -11 | 17 |
| Iceland | 06.04.2020 | 16.03.2020 | 23.03.2020 | 54 | -21 |  |
| Ireland | 23.03.2020 | 13.03.2020 | 09.04.2020 | 91 | -10 | 17 |
| Italy | 24.02.2020 | 22.02.2020 | 20.03.2020 | 92 | -2 | 21 |
| Luxembourg | 21.03.2020 | 13.03.2020 | 22.03.2020 | 80 | -8 |  |
| Netherlands | 13.03.2020 | 12.03.2020 | 05.04.2020 | 80 | -1 | 12 |
| Norway | 18.03.2020 | 12.03.2020 | 23.03.2020 | 70 | -6 | 12 |
| Poland | 22.03.2020 | 12.03.2020 | 05.04.2020 | 81 | -10 | 20 |
| Portugal | 20.03.2020 | 16.03.2020 | 29.03.2020 | 82 | -4 | 11 |
| Romania | 23.03.2020 | 09.03.2020 | 09.04.2020 | 87 | -14 | 28 |
| Serbia | 28.03.2020 | 15.03.2020 | 11.04.2020 | 100 | -13 | 12 |
| Slovakia | 15.04.2020 | 10.03.2020 | 13.04.2020 | 87 | -36 |  |
| Slovenia | 26.03.2020 | 16.03.2020 | 24.03.2020 | 79 | -10 |  |
| Spain | 07.03.2020 | 10.03.2020 | 25.03.2020 | 72 | 3 | 12 |
| Sweden | 16.03.2020 | 29.03.2020 | 19.04.2020 | 46 | 13 | 23 |
| Switzerland | 13.03.2020 | 13.03.2020 | 21.03.2020 | 77 | 0 | 11 |
| United Kingdom | 10.03.2020 | 22.03.2020 | 08.04.2020 | 76 | 12 | 21 |

Supplementary Table 2: AIC for test to death period using gamma, Weibull and lognormal distributions

| Distribution | Gamma | Weibull | Lognormal |
| --- | --- | --- | --- |
| AIC | **8997** | 9026 | 9085 |

# References

Cori, Anne, Neil M. Ferguson, Christophe Fraser, et Simon Cauchemez. 2013. « A New Framework and Software to Estimate Time-Varying Reproduction Numbers During Epidemics ». *American Journal of Epidemiology* 178 (9): 1505‑12. https://doi.org/10/f5gwtn.
